# Using the Timmer Scale to Standardize Pediatric Dentistry Residents’ Scientific Appraisal Skills

**DOI:** 10.15766/mep_2374-8265.11101

**Published:** 2021-02-12

**Authors:** Samah Omar, Janet Bauer, Afsaneh Matin, Amanjyot Bians, Jung-Wei Chen, Monica Ocampo

**Affiliations:** 1 Associate Professor, Pediatric Dentistry Department, School of Dentistry, Loma Linda University; 2 Professor Emerita, UCLA School of Dentistry and School of Dentistry, Loma Linda University; 3 Assistant Professor, Pediatric Dentistry Department, School of Dentistry, Loma Linda University; 4 Former Volunteer Research Assistant, Center of Dental Research, School of Dentistry, Loma Linda University; 5 Professor and Program Director, Advanced Specialty Education Program of Pediatric Dentistry, School of Dentistry, Loma Linda University

**Keywords:** Quality Assessment, Journal Club, Evidence-Based Practice, Pediatric Dentistry

## Abstract

**Introduction:**

There is no consensus on the best methodology to apply evidence-based practice principles to develop a systematic approach to improve critical appraisal or research design evaluation skills in advanced education journal clubs.

**Methods:**

We implemented a tool-based approach for our pediatric dentistry residents’ journal club centered on the use of a study quality assessment tool, the Timmer scale. The tool consisted of 19 standard questions that evaluated the research methodology, data collection, statistical analysis, and reporting of the findings of each article. Learners first underwent a 4-hour training session on study quality assessment. They were then assigned to read articles from monthly issues of core journals and appraised the quality of each article using the Timmer scale and submitted their scores in advance of the group session. Then, during a 1-hour journal club, the group came to a consensus on the Timmer scale score, and group and individual scores were compared to the course director's scores as prompts for feedback and further discussion.

**Results:**

Over 3 years, 24 pediatric dentistry residents participated in the course. A noticeable improvement in the pediatric dentistry residents’ performance was noticed, with the discrepancy between their scores and group scores improving over time.

**Discussion:**

Using a quality assessment tool in journal clubs appeared to improve the residents’ ability to critically assess articles in a systematic way. Additionally, the tool was useful for assessing residents’ performance over time.

## Educational Objectives

By the end of this activity, participants will be able to:

1. Develop a systematic approach for critically appraising articles based on simple research design principles.
2. Evaluate the literature using a quality assessment tool (Timmer scale).
3. Improve critical thinking skills and article appraisal abilities.
4. Develop evidence-based practice principles in a journal club setup.

## Introduction

Journal club seminars are typically a forum intended to review, discuss, and evaluate the professional literature from a specific discipline. Historically, pedagogical studies looking at the effectiveness of the journal club approach in health care education have mainly focused on the advantages, periodicity, and article selection.^1–9^ Numerous models have been published on how to establish and conduct journal clubs.^1,3–5,8–11^ However, none of these presented a specific validated tool or applied evidence-based practice (EBP) to develop a systematic approach to critical appraisal or research design evaluation. More recent studies of journal club pedagogy focused on the assessment of research articles using evidence-based methodologies.^11,12^ Ahmadi et al. performed a systematic review and found that while journal clubs seem to be the preferred way of teaching critical appraisal skills and evaluating the literature, there is a lack of consistency in terms of the appropriate format of the journal clubs and their effectiveness in determining the quality of individual articles.^10^ To the best of our knowledge, this is the first presentation of a systematic approach to teaching journal club using a validated tool.

The Timmer scale^13^ is an assessment tool developed to evaluate article quality. It has shown evidence of validity for assessing the quality of abstracts submitted to professional research conferences. The tool consists of 19 standard questions that evaluate the research methodology, data collection, statistical analysis, and reporting of the findings. Because it uses a scale rather than a checklist and articulates the domains in which a research article can be systematically analyzed, we decided to use it with pediatric dentistry residents to practice the skill of literature appraisal.

Since the 1970s, multiple assessment instruments have been introduced to provide methodology for critical appraisal in systematic reviews of the professional literature, including: Consolidated Standards of Reporting Trials (CONSORT),^14^ Timmer,^13^ Jadad,^15^ Assessment of Multiple Systematic Reviews (AMSTAR),^16^ Revised Assessment of Multiple Systematic Reviews (R-AMSTAR),^17^ Preferred Reporting Items for Systematic Reviews and Meta-Analyses (PRIMSA),^18^ and Grading of Recommendations Assessment, Development and Evaluation (GRADE).^19^ While most of these tools have multiple forms for different study designs like Oxford and the *Journal of the American Medical Association*,^21–25^ Timmer, which has been validated and determined to be reliable for assessing research design methodology, has one form with standardized questions that critique the design, statistical methodology, and reporting of data. The specific study design is factored in using a specific formula that takes into account the randomization. It also has a numerical scale that allowed ranking of articles for quality. As a result, it is easier to train and calibrate residents to use the Timmer scale.

## Methods

In 2014 we implemented a new systematic approach to evaluating and critiquing the articles discussed in our weekly journal club course using the Timmer scale.^13^ Journal club attendance was mandatory for all residents enrolled in the program and optional for dental students, staff, and the other program faculty. The discussion was usually facilitated by the course director with input from other attendees. The course director had prior training in principles of EBP and article review methodologies. A 4-hour training session was provided to all residents prior to the beginning of the journal club course (Appendices A and B). This training session introduced the residents to the objectives of EBP, basic research design principles, and trained the participants to use the quality assessment tool (Timmer scale^13^). During this session, the residents practiced scoring a random article using the Timmer scale, with the course director's guidance. The residents were also provided with a list of scientific articles (Appendix G) related to implementing EBP in dentistry to review before the training session.

Three or four different articles were read and assessed for every weekly session. Selected articles were acquired from the most current *Pediatric Dentistry* journal volume. The assigned article schedule was posted every 3 months or as a new journal issue was published. A minimum of 10–14 days was given to the residents to review and score the articles. Only clinical trials and systematic reviews were scored; in-vitro studies, literature reviews, and case reports were not scored. Unscored articles were included in the general discussion and were evaluated for the clinical and scientific relevance.

After reading an article, residents calculated a summary score (SS) using the Quality Assessment Score (QAS) Sheet (Appendix C) and the Study Design and Total Possible Points Form (Appendix D) which were simplified color-coded forms adapted from the original 19-question Timmer tool^13^ by the course director with permission from the creators. Residents first completed the QAS sheet. Each question was scored as *completely applies* (2 points), *partially applies* (1 point), or *does not apply* (0 points). The only exception was question 7, which was scored as 2 if the study was randomized and 0 if it was not. After each question was scored, the scores were summed for the QAS, which ranged from 0 to 38. After the QAS was determined, a study design score (SDS) and total possible points (TPP), assigned to the specific study design, were identified from the reported research methodology (Appendix D).All scores were then compiled to calculate an SS using the following formulas: For studies without randomized sample subjects, SS = (QAS + SDS)/TPP. For studies with randomized sample subjects, SS = (QAS + SDS + 1)/TPP. Calculated SS was a fraction number between .09 (if QAS = 0) and 1 (if QAS = 38); the closer the number to 1, the higher the quality of the study. In addition to the scoring, the residents were asked to describe the strengths and weaknesses of each article in a separate form (Appendix E). All article scores were sent to the course director 2 days prior to the journal club meeting for review. These scores were used for evaluating each resident’s performance.

Calculated SS was a fraction number between .09 (if QAS = 0) and 1 (if QAS = 38); the closer the number to 1, the higher the quality of the study. In addition to the scoring, the residents were asked to describe the strengths and weaknesses of each article in a separate form (Appendix E). All article scores were sent to the course director 2 days prior to the journal club meeting for review. These scores were used for evaluating each resident's performance.

**Figure 1.**
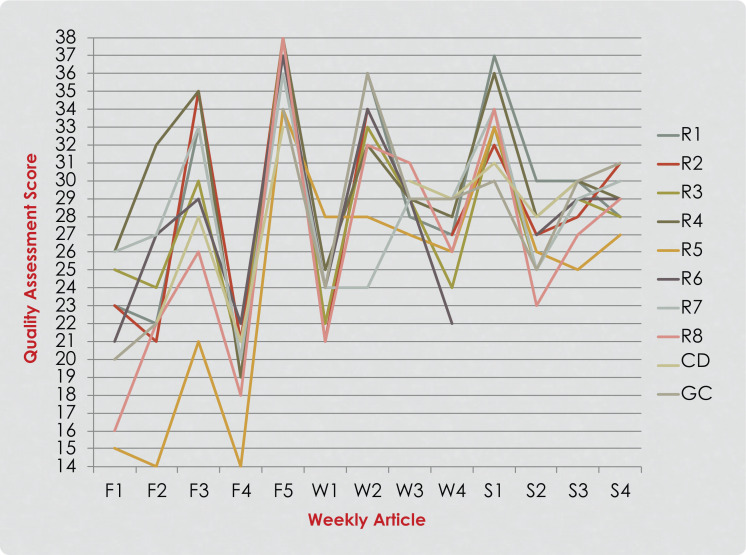
Residents’ (R1-R8), course director (CD), and group consensus (GC) quality assessment scores of articles read during the first year of the implementation. Five articles were read and rated in the fall quarter (F1-F5), four in the winter quarter (W1-W4), and four in the spring quarter (S1-S4).

**Figure 2.**
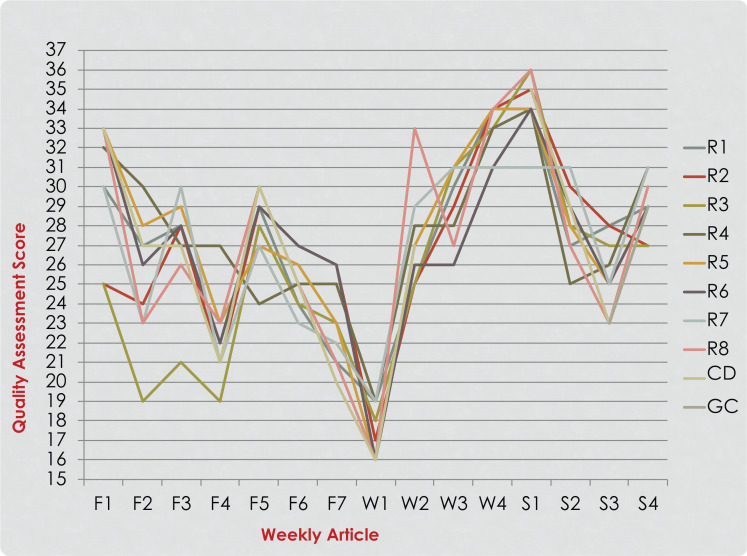
Residents’ (R1-R8), course director (CD), and group consensus (GC) quality assessment scores of articles read during the second year of the implementation. Seven articles were read and rated in the fall quarter (F1-F7), four in the winter quarter (W1-W4), and four in the spring quarter (S1-S4).

**Figure 3.**
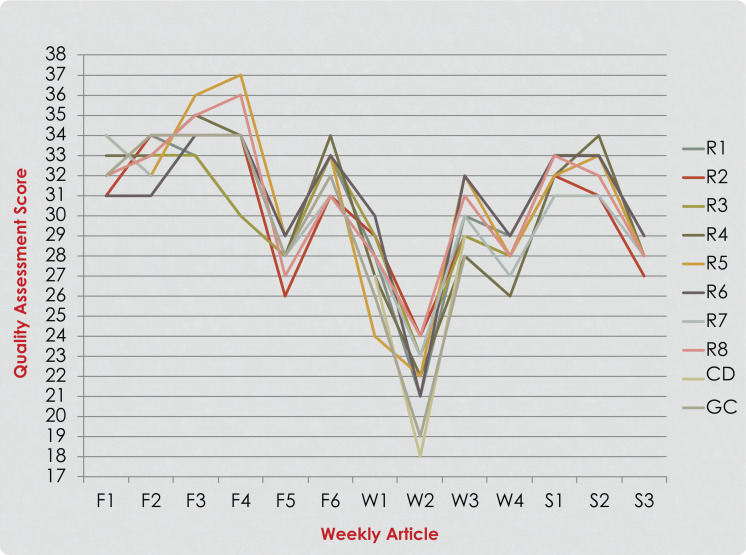
Residents’ (R1-R8), course director (CD), and group consensus (GC) quality assessment scores of articles read during the third year of the implementation. Six articles were read and rated in the fall quarter (F1-F6), four in the winter quarter (W1-W4) and three in the spring quarter (S1-S3).

**Figure 4.**
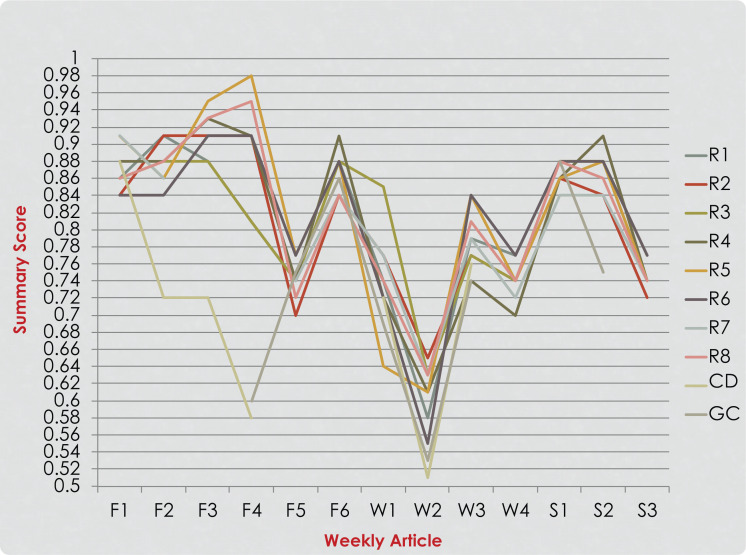
Residents’ (R1-R8), course director (F1), and group consensus (GC) summary scores (SS) of articles read during the third year of the implementation. Six articles were read and rated in the fall quarter (F1-F6), four in the winter quarter (W1-W4) and three in the spring quarter (S1-S3).

The course director independently scored the assigned articles prior to the seminar and summarized the research design, QAS, SDS, TPP, and SS for each article using a summary sheet. The course director compiled the scores from all residents, faculty, and the group score, and presented them to the class in a graph format (Figures 1–4) weekly. Each resident was assigned a random number at the beginning of the year so they could track their individual performance when the graph was shared in class. Immediate feedback was given anonymously to the whole group during the session and each article was discussed and critiqued by all participants, facilitated by the course director. After these discussions, the group determined a consensus group score for SS and QAS for each article.

At the end of every quarter, all participants filled out a course evaluation survey (Appendix F). When major misinterpretations of the critical appraisal process were identified, relevant reading assignments were used in 20–30 minutes remedial topic-focused training sessions (Appendix G). Information extracted from the survey along with the session's feedback were used to modify and improve the precourse training session. Based on this information, we assigned readings to be reviewed before the training session (Appendix G).

The performance of residents was evaluated over a 3-year period to determine the impact of implementing the Timmer scale^13^ on our pediatric dentistry residents’ ability to assess articles in a journal club environment. This was done based on the residents’ performance, by comparing their scores to course director and group consensus scores over time. Secondarily, the authors evaluated the applicability of the Timmer instrument as a teaching tool to assess teaching outcomes and improve evidence-based instructional material. We evaluated the process by comparing the residents’ scores over the 3-year period.

## Results

Eight pediatric dentistry residents (four first-year and four second-year) participated in journal club course every year over the 3 year study period (24 participants in total). The participants’ QAS scores for each year of implementation are presented in Figures 1–3. A noticeable improvement in the residents’ critical appraisal skills was evidenced by the decrease in the discrepancy between their scores and group consensus scores over the course of each year of implementation. When looking at the residents’ scores in the fall quarter, greater variation in their scores was noticed. Residents’ performance improved over time as the scores became closer in value to each other and to the group consensus scores as they reached the end of the spring quarter (Figures 1–3). Discrepancies between the QAS graph (Figure 3) and the SS graph (Figure 4) indicated mistakes in study design identification or inaccurate SS formula calculation. When comparing residents’ performance in different years, we found that there was lower discrepancy among the scores in the third year of implementation (Figure 3) compared to the first year of implementation (Figure 1).

## Discussion

The utilization of an established quality assessment tool and structured worksheets improved residents’ critical appraisal skills in a journal club setting. At the beginning of the fall quarter, residents were able to score the articles fairly, but did not show full understanding of the concepts demonstrated by the variability in the residents’ scores. At the end of the year, resident scores showed improvement by scoring near the group consensus score. Before the implementation of this tool-based approach, the residents were using their own subjective judgments that were based on arbitrary evaluations influenced by their diverse backgrounds and experiences rather than a systematic and scientific approach. The structured Timmer scale^13^ helped residents focus on specific elements of the research methodology and data interpretation, all of which improved their critical appraisal skills.

In recent years, many advanced educational and training programs have adopted different methods and modules to integrate critical appraisal skills in their curriculum using EBP methodology.^1,3–5,8–11^ However, no consensus on a standard model has emerged. Although a different approach, our findings were consistent with Moher,^4^ who found that an evidence-based medicine curriculum improved residents’ performance after attending at least six journal club sessions. Our results also add to the current body of literature on evaluating assessment instruments for implementation into journal club settings.^7,12,26–31^ Atzema reports that using a validated and well-structured assessment instrument may enhance the residents’ assessment skills of research design methodology and reporting of findings.^27^ It may also give residents confidence to read research articles and extract clinically relevant quality evidence that will help them improve clinical care.

When comparing overall performance over several years, we noticed less variation among residents in the third year (Figure 3) compared to the first year (Figure 1). This demonstrated improvement in the instructional course over the years. This also indicated that critical appraisal and quality assessment in research design and methodology is a process that is acquired over a period of time and highlighted the importance of continuity and consistency of training. This supported the findings of earlier research in which authors questioned the retention of EBP knowledge and skills after graduation from training programs and suggested that skills and knowledge might be lost if not updated by continuous training or engagement in local journal clubs.^8,32–34^

Plotting all data in a graph prior to class helped the course director identify resident performance outliers or discrepancies between the residents’ QAS or SS and the group scores. Discrepancies indicated errors in identifying study design, randomization, or SS calculation errors. Sharing the progress report in the format of a color-coded graph every session had a positive influence on participant's motivation and allowed for quick visualization of individual performance over time and identification of errors and mistakes. Discrepancies between QAS and SS graphs indicated errors in study design identification or inaccurate SS formula calculation. For example, after examining the graphs in Figures 3 and 4, it was readily apparent that two residents (R5 and R7) had discrepancies in QAS and SS of articles S3 and S4, indicating an error that needed evaluation and discussion. The same was true for R7 relative to article W3. The resident made a mistake in identifying the research design, affecting the SS calculation. This error in the research design was discussed with the group to clarify any confusion. Based on residents’ feedback from course evaluations (Appendix F) and identification of common challenges in critical appraisal, modifications were implemented in the following year's introductory course as described in the Methods.

Limitations of this educational approach included using a convenience sample of articles for the assessment. Thus, at each time period, all assessed articles differed in content, rendering it difficult to standardize. Our advanced training program is a small 2-year program, resulting in a small participant size of eight residents at all times. The heterogenicity of the participants, along with the small sample size, made it hard to validate our observations with statistical analysis. Longer-term studies with larger sample sizes would be beneficial to further support our findings.

We concluded that incorporating a validated assessment tool like the Timmer scale into journal club enhanced residents’ QAS and improved their approach to evaluating published research. The Timmer assessment tool^13^ was useful to monitor and evaluate residents’ performance in assessing quality of articles when compared to group consensus in a journal club setting. It is also a valuable teaching tool to improve critical appraisal and evidence-based teaching in residency programs.

## Appendices

Introductory Course Material (EBP).pptxJournal Club Course Introduction.pptxQuality Assessment Score Sheet.docxStudy Design and Total Possible Points Form.docxArticles Evaluation Form.docxCourse Evaluation Form.docxPreclass and Remediation Reading Assignments.docx
All appendices are peer reviewed as integral parts of the Original Publication.
