# Supplementary material for: Using the Timmer Scale to Standardize Pediatric Dentistry Residents’ Scientific Appraisal Skills
Source: MedEdPORTAL. 2021 Feb 12;17:11101. doi: 10.15766/mep_2374-8265.11101 (PMC7880256; doi:10.15766/mep_2374-8265.11101)
Supplement: Supplementary file 1 — Introductory Course Material (EBP).pptxJournal Club Course Introduction.pptxQuality Assessment Score Sheet.docxStudy Design and Total Possible Points Form.docxArticles Evaluation Form.docxCourse Evaluation Form.docxPreclass and Remediation Reading Assignments.docx [file mep_2374-8265.11101-s001.zip › A. Introductory Course Material (EBP).pptx]

## Slide 1
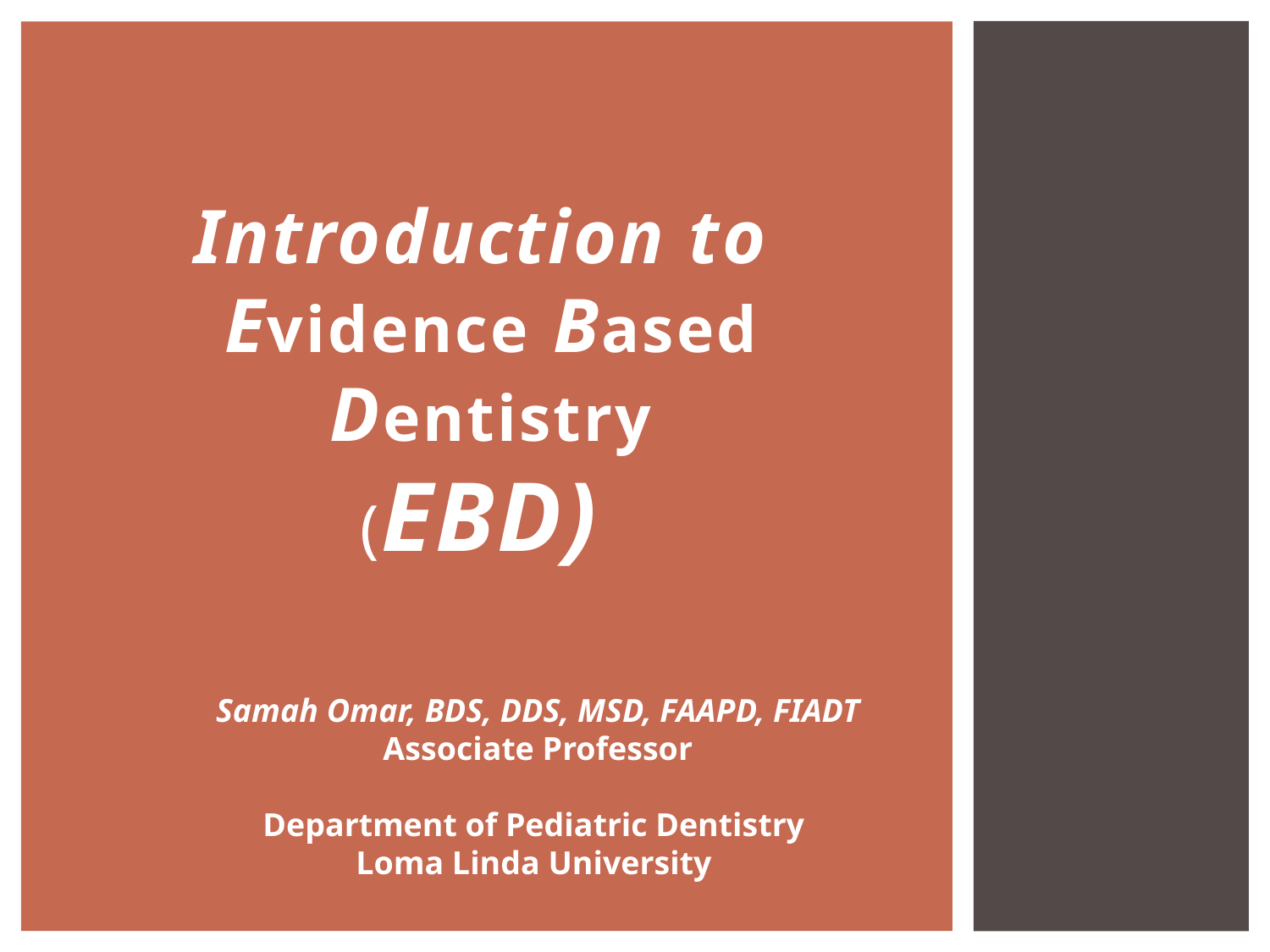

# Introduction to Evidence Based Dentistry (EBD)
Samah Omar, BDS, DDS, MSD, FAAPD, FIADT
Associate Professor
Department of Pediatric Dentistry
Loma Linda University

## Slide 2
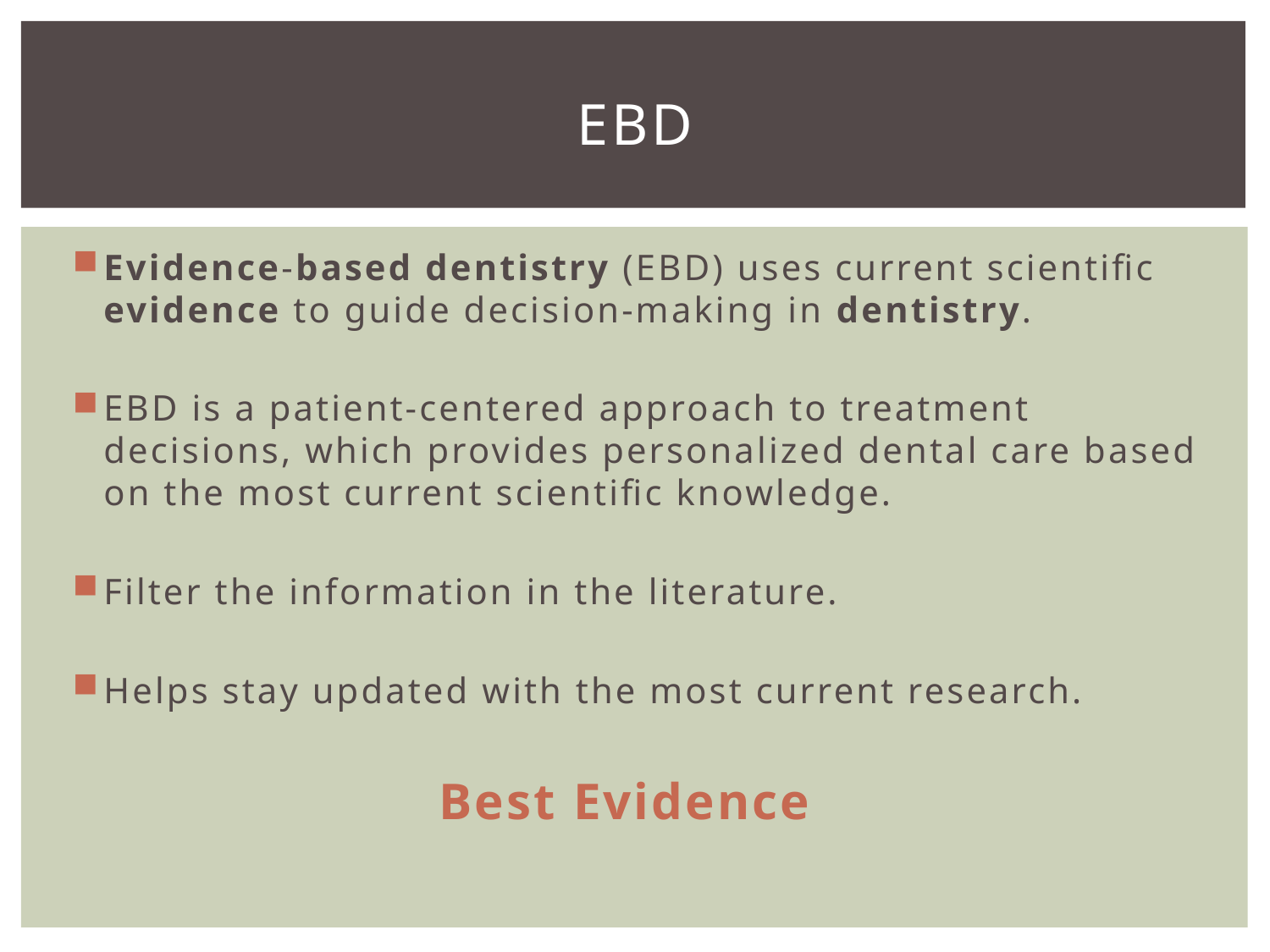

# EBD
Evidence-based dentistry (EBD) uses current scientific evidence to guide decision-making in dentistry.
EBD is a patient-centered approach to treatment decisions, which provides personalized dental care based on the most current scientific knowledge.
Filter the information in the literature.
Helps stay updated with the most current research.
Best Evidence

## Slide 3
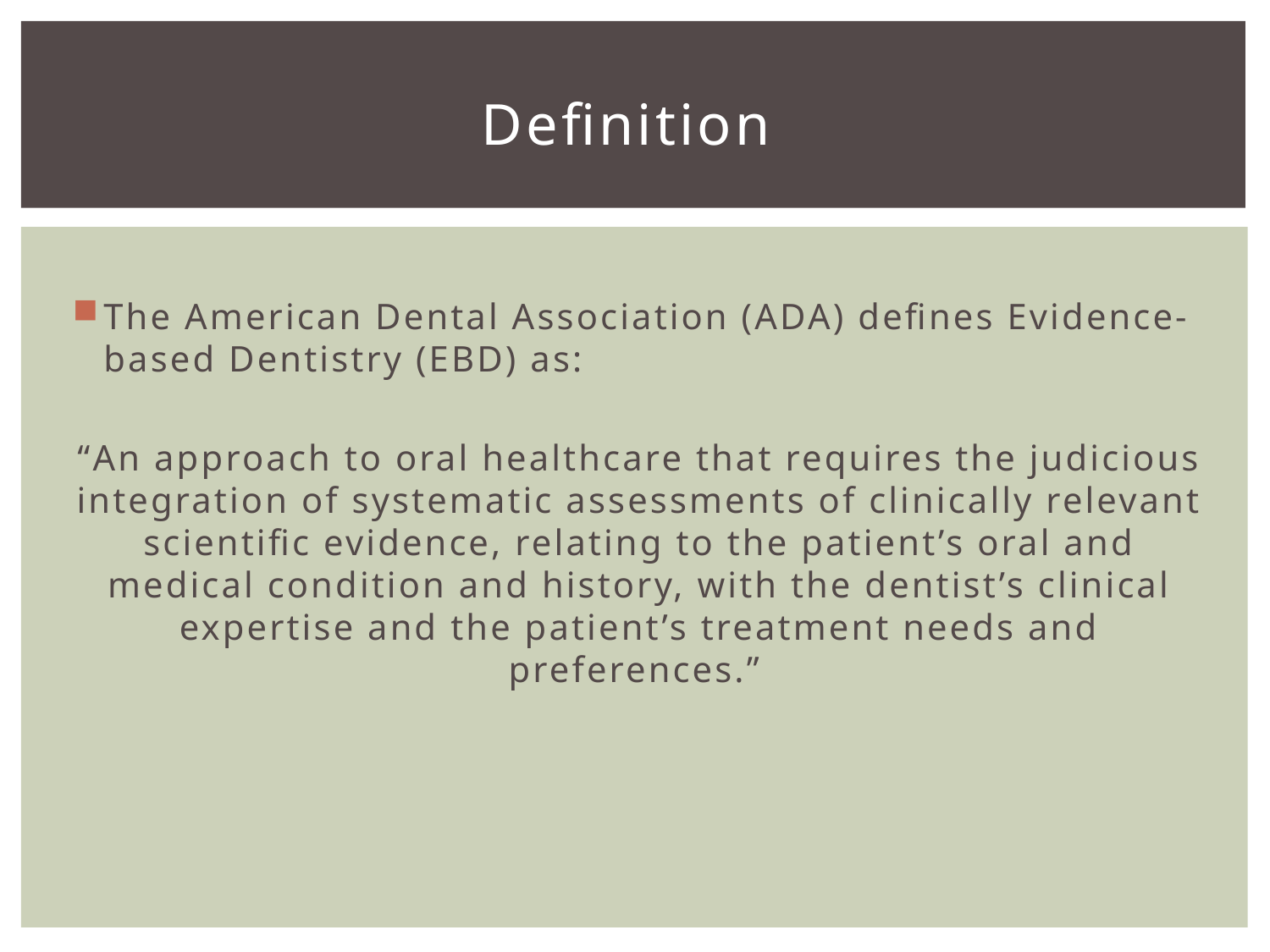

# Definition
The American Dental Association (ADA) defines Evidence-based Dentistry (EBD) as:
“An approach to oral healthcare that requires the judicious integration of systematic assessments of clinically relevant scientific evidence, relating to the patient’s oral and medical condition and history, with the dentist’s clinical expertise and the patient’s treatment needs and preferences.”

## Slide 4
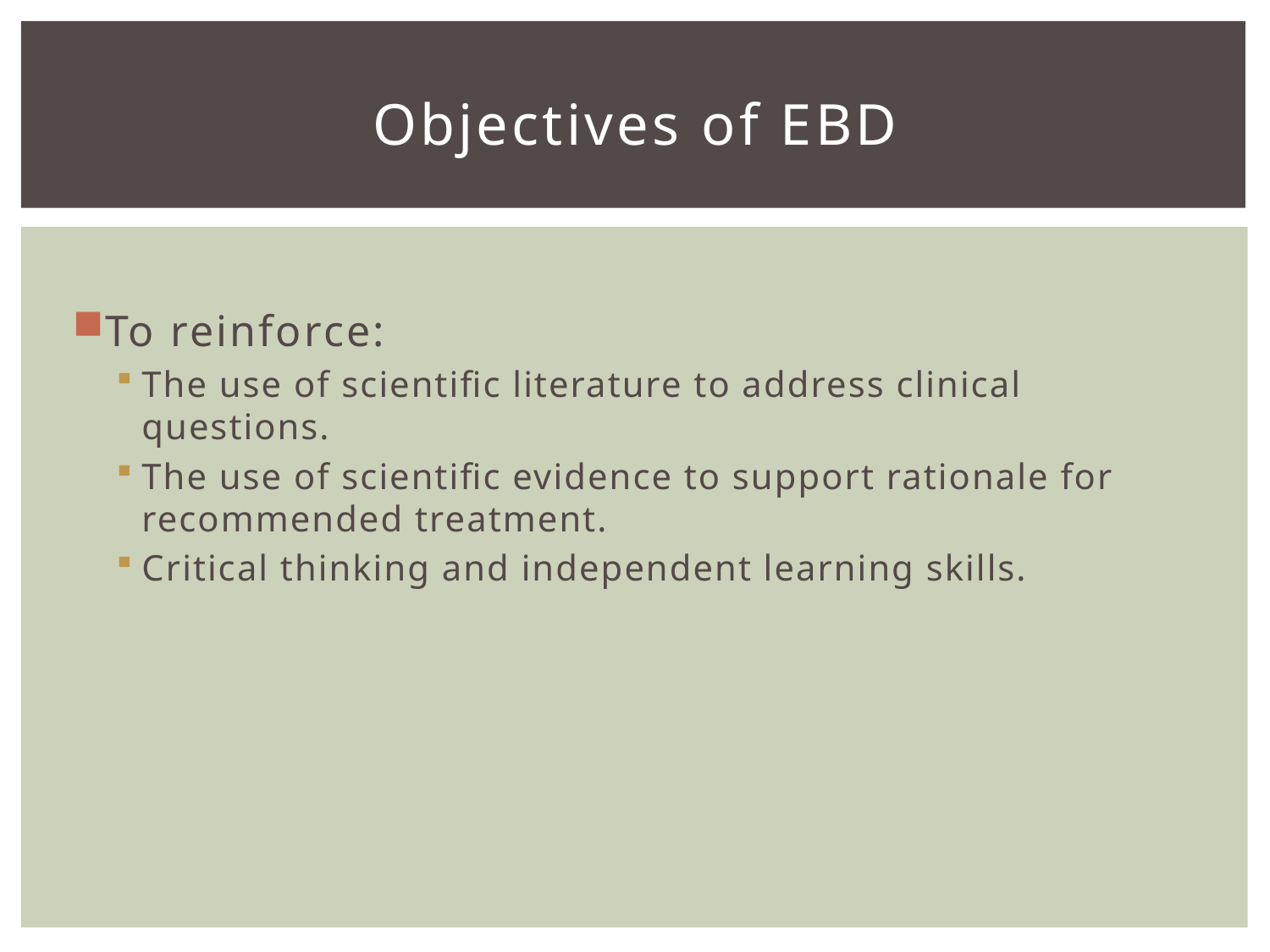

# Objectives of EBD
To reinforce:
The use of scientific literature to address clinical questions.
The use of scientific evidence to support rationale for recommended treatment.
Critical thinking and independent learning skills.

## Slide 5
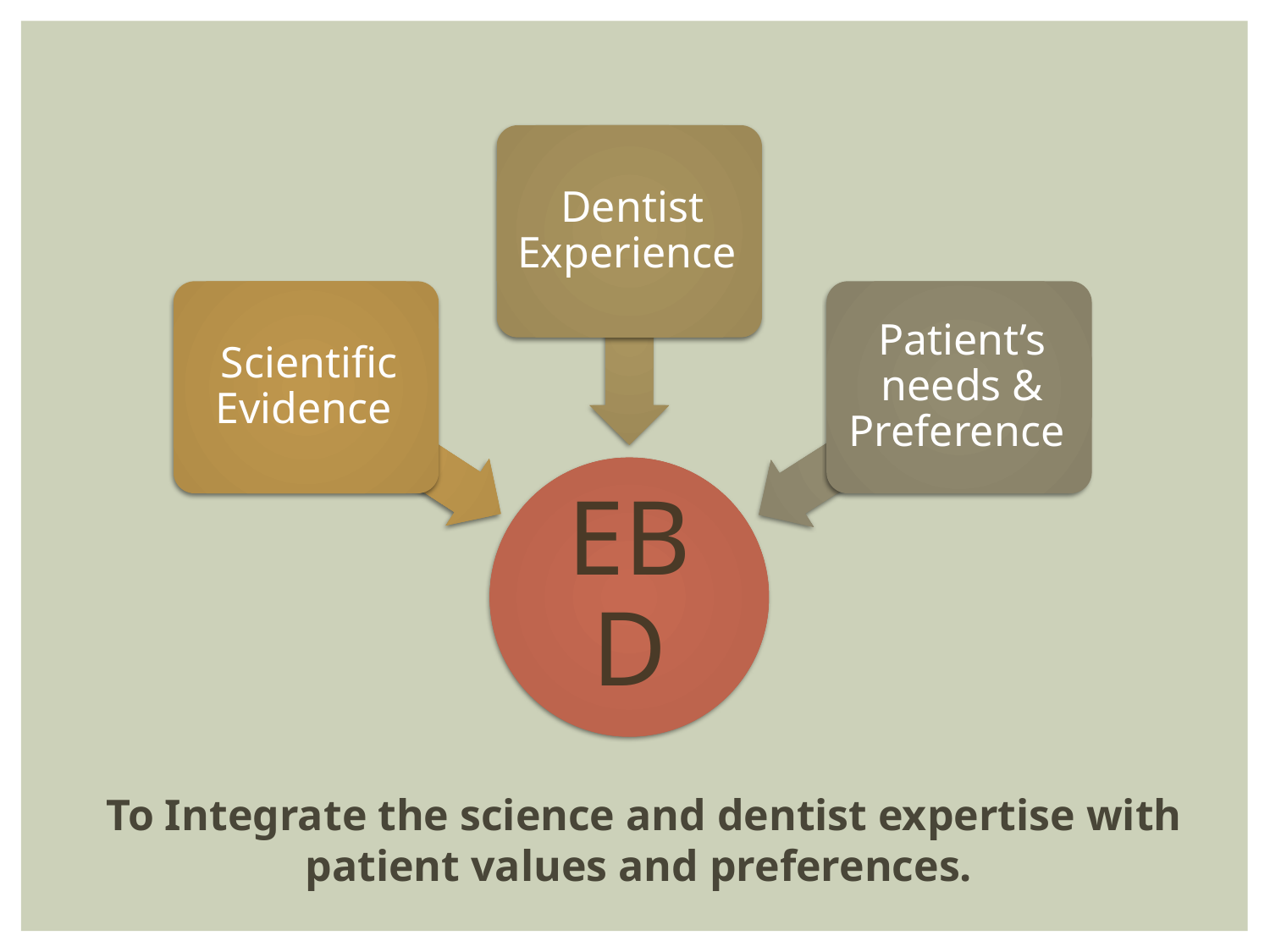

To Integrate the science and dentist expertise with patient values and preferences.

## Slide 6
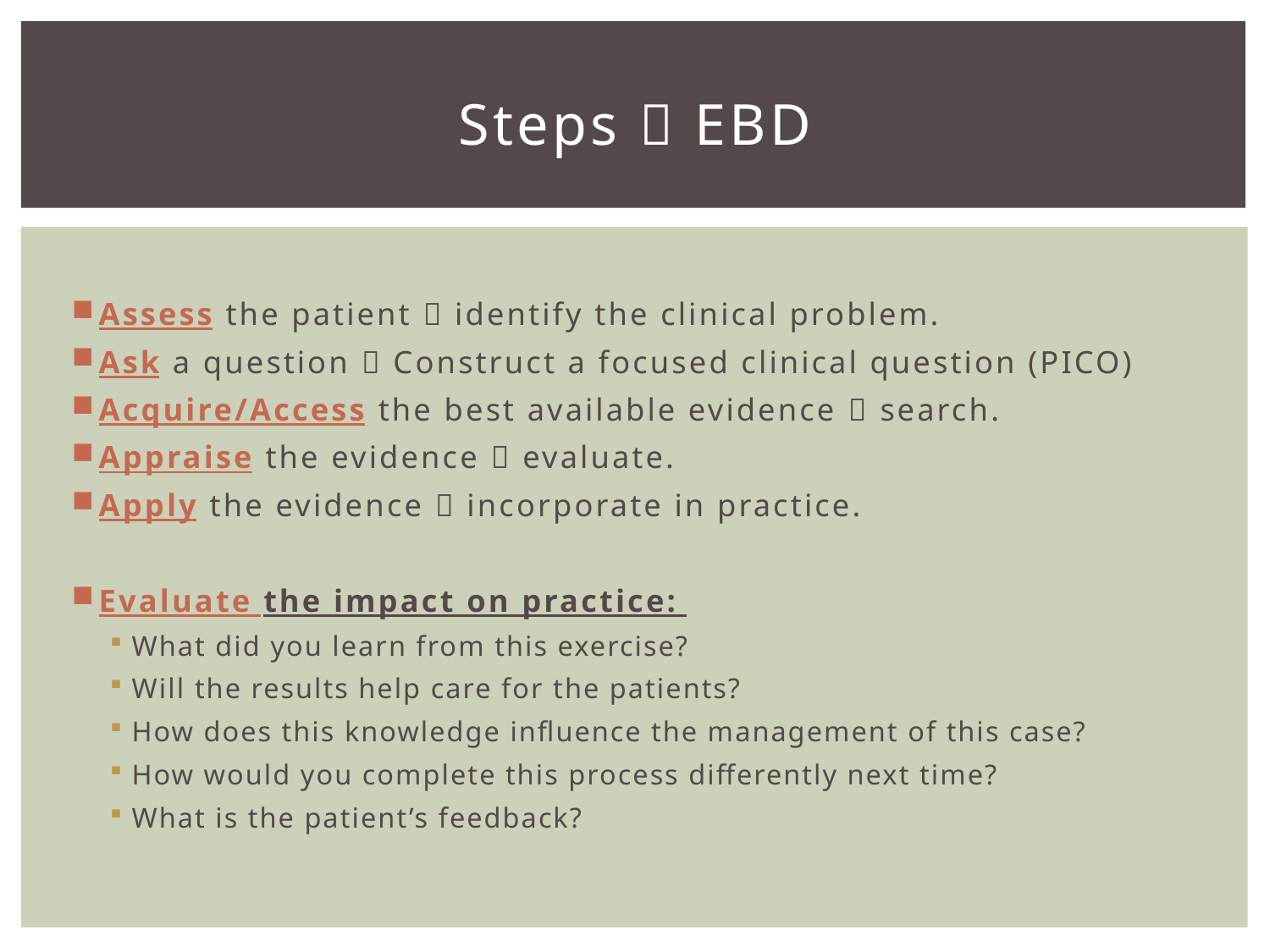

# Steps  EBD
Assess the patient  identify the clinical problem.
Ask a question  Construct a focused clinical question (PICO)
Acquire/Access the best available evidence  search.
Appraise the evidence  evaluate.
Apply the evidence  incorporate in practice.
Evaluate the impact on practice:
What did you learn from this exercise?
Will the results help care for the patients?
How does this knowledge influence the management of this case?
How would you complete this process differently next time?
What is the patient’s feedback?

## Slide 7
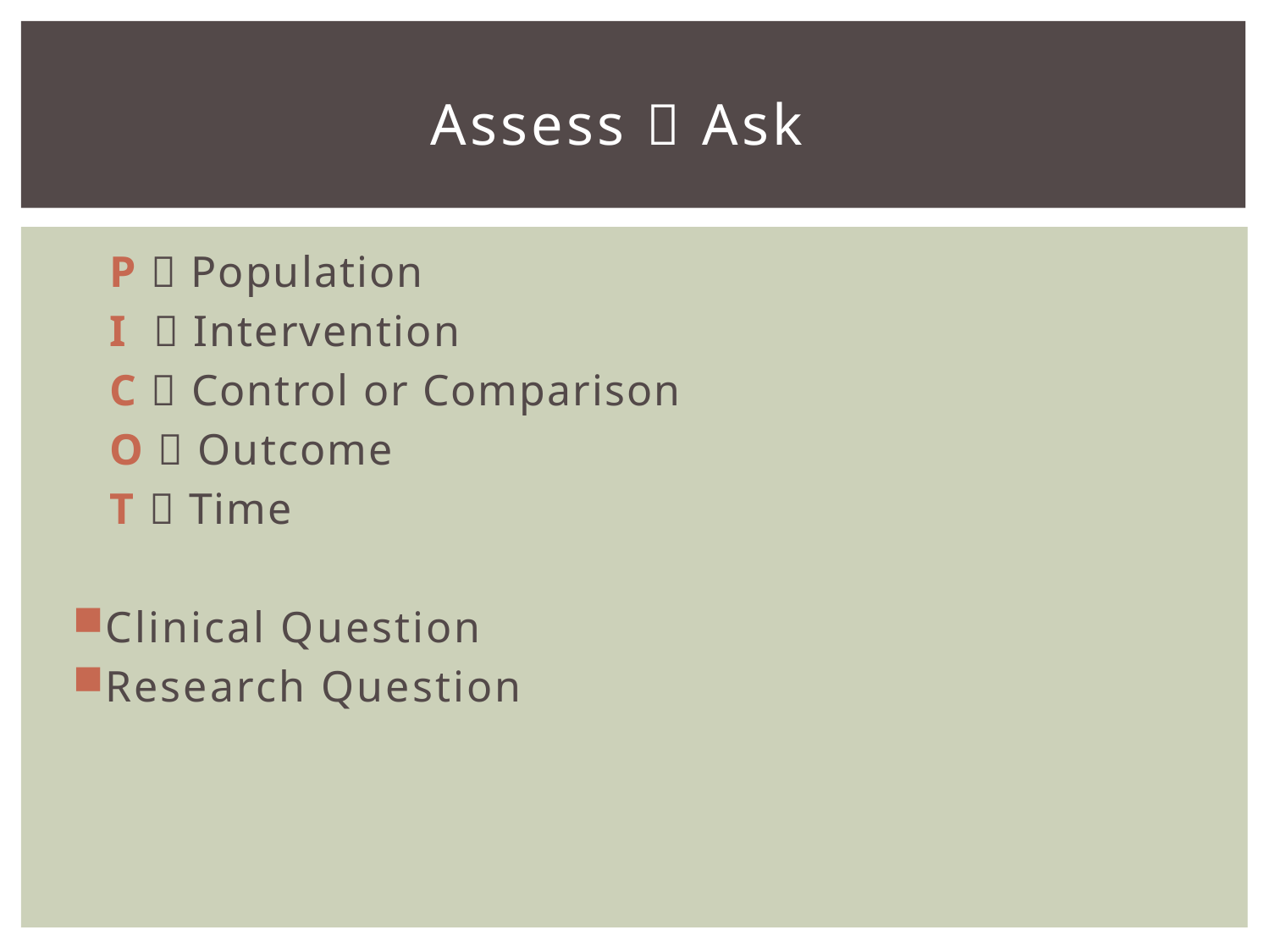

# Assess  Ask
P  Population
I  Intervention
C  Control or Comparison
O  Outcome
T  Time
Clinical Question
Research Question

## Slide 8
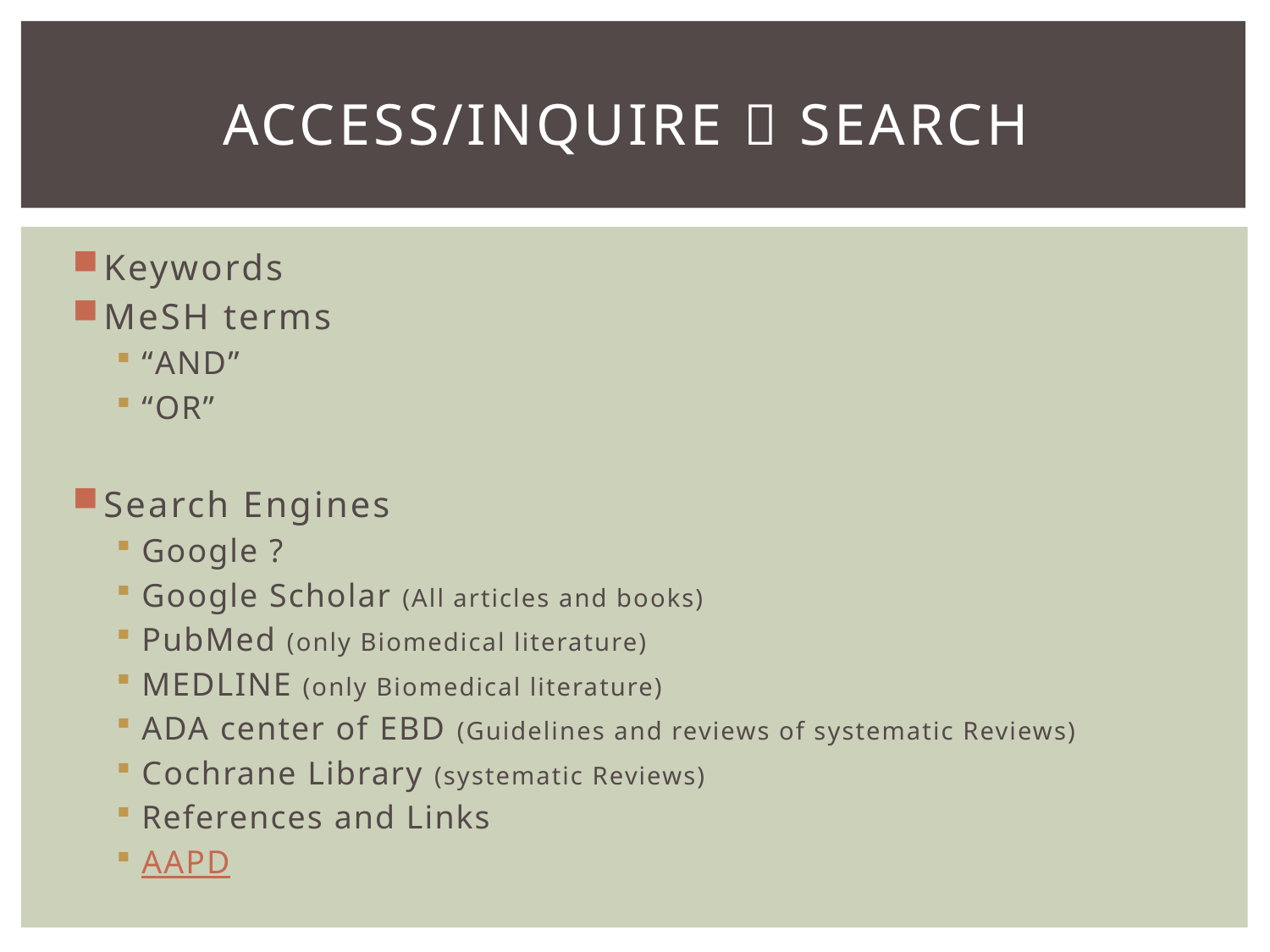

# Access/Inquire  Search
Keywords
MeSH terms
“AND”
“OR”
Search Engines
Google ?
Google Scholar (All articles and books)
PubMed (only Biomedical literature)
MEDLINE (only Biomedical literature)
ADA center of EBD (Guidelines and reviews of systematic Reviews)
Cochrane Library (systematic Reviews)
References and Links
AAPD

## Slide 9
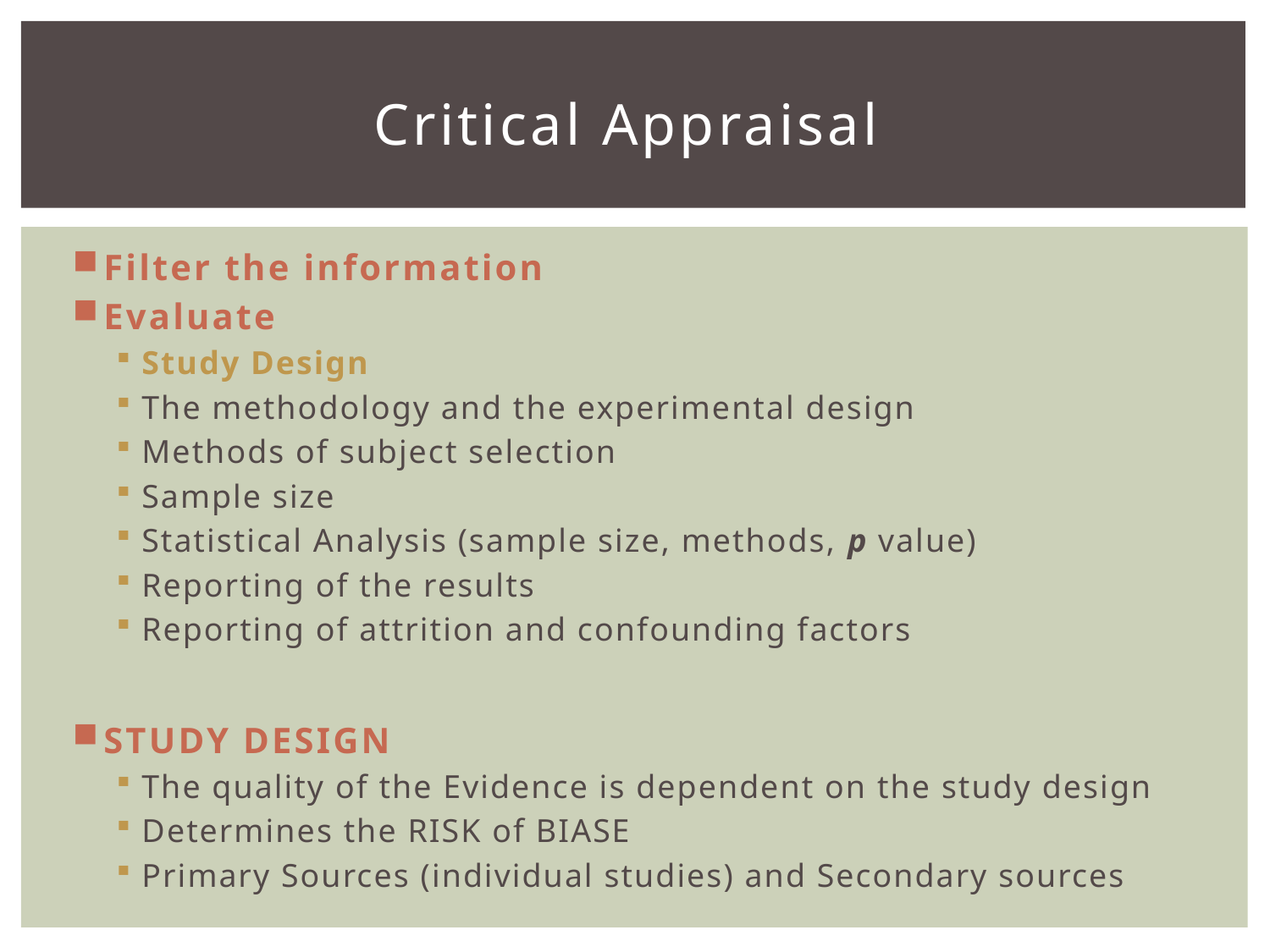

# Critical Appraisal
Filter the information
Evaluate
Study Design
The methodology and the experimental design
Methods of subject selection
Sample size
Statistical Analysis (sample size, methods, p value)
Reporting of the results
Reporting of attrition and confounding factors
STUDY DESIGN
The quality of the Evidence is dependent on the study design
Determines the RISK of BIASE
Primary Sources (individual studies) and Secondary sources

## Slide 10
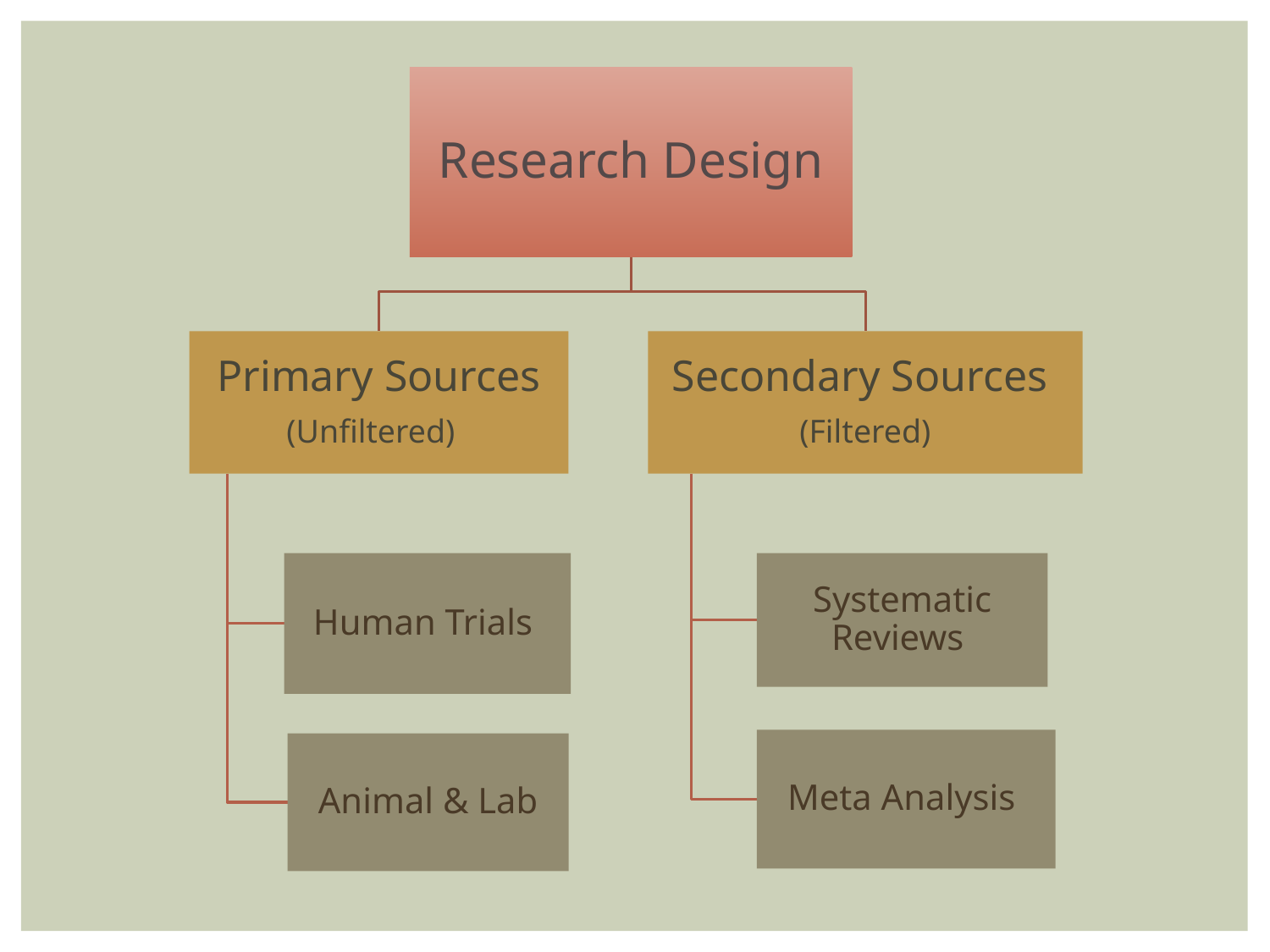

## Slide 11
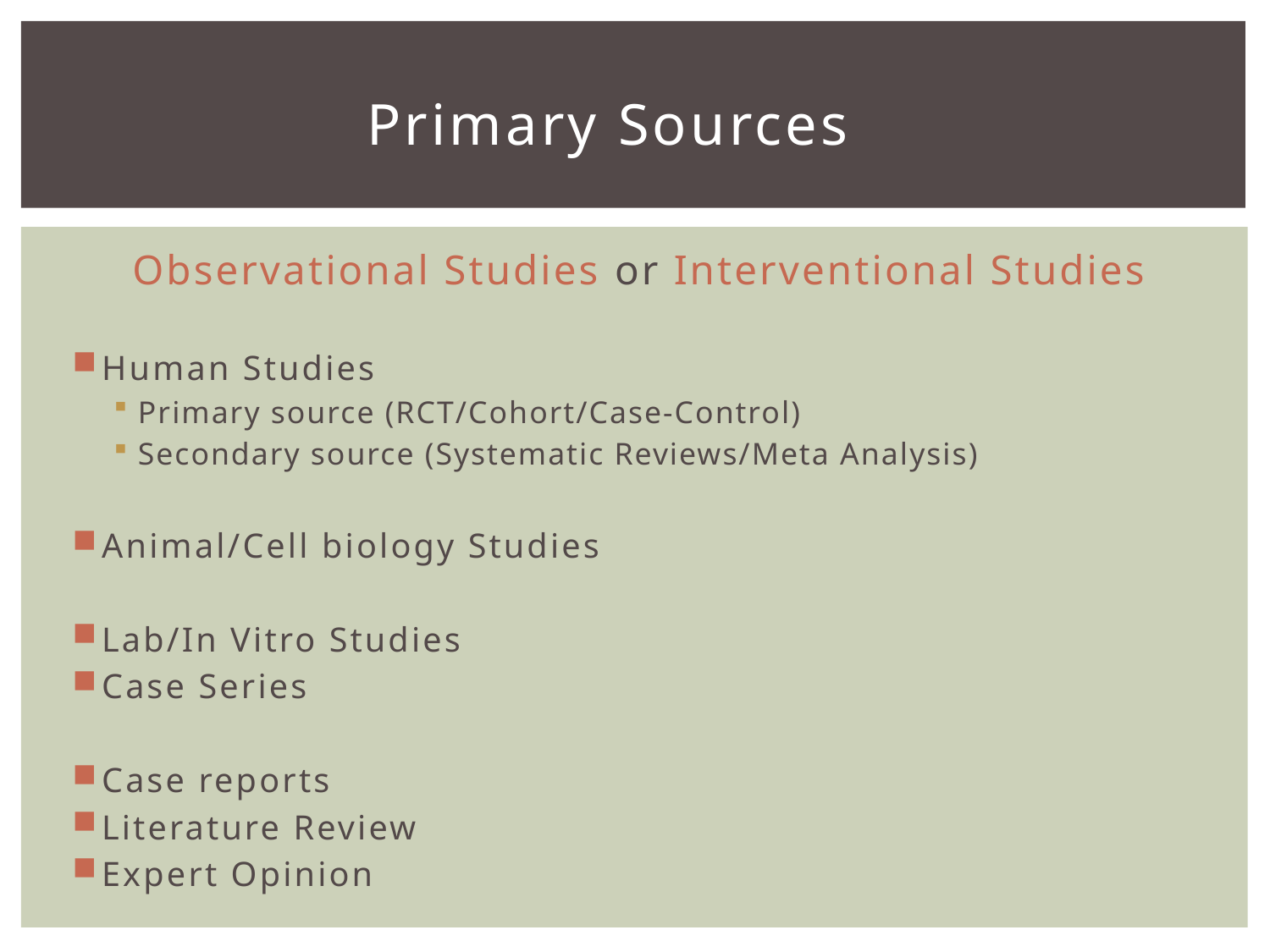

# Primary Sources
 Observational Studies or Interventional Studies
Human Studies
Primary source (RCT/Cohort/Case-Control)
Secondary source (Systematic Reviews/Meta Analysis)
Animal/Cell biology Studies
Lab/In Vitro Studies
Case Series
Case reports
Literature Review
Expert Opinion

## Slide 12
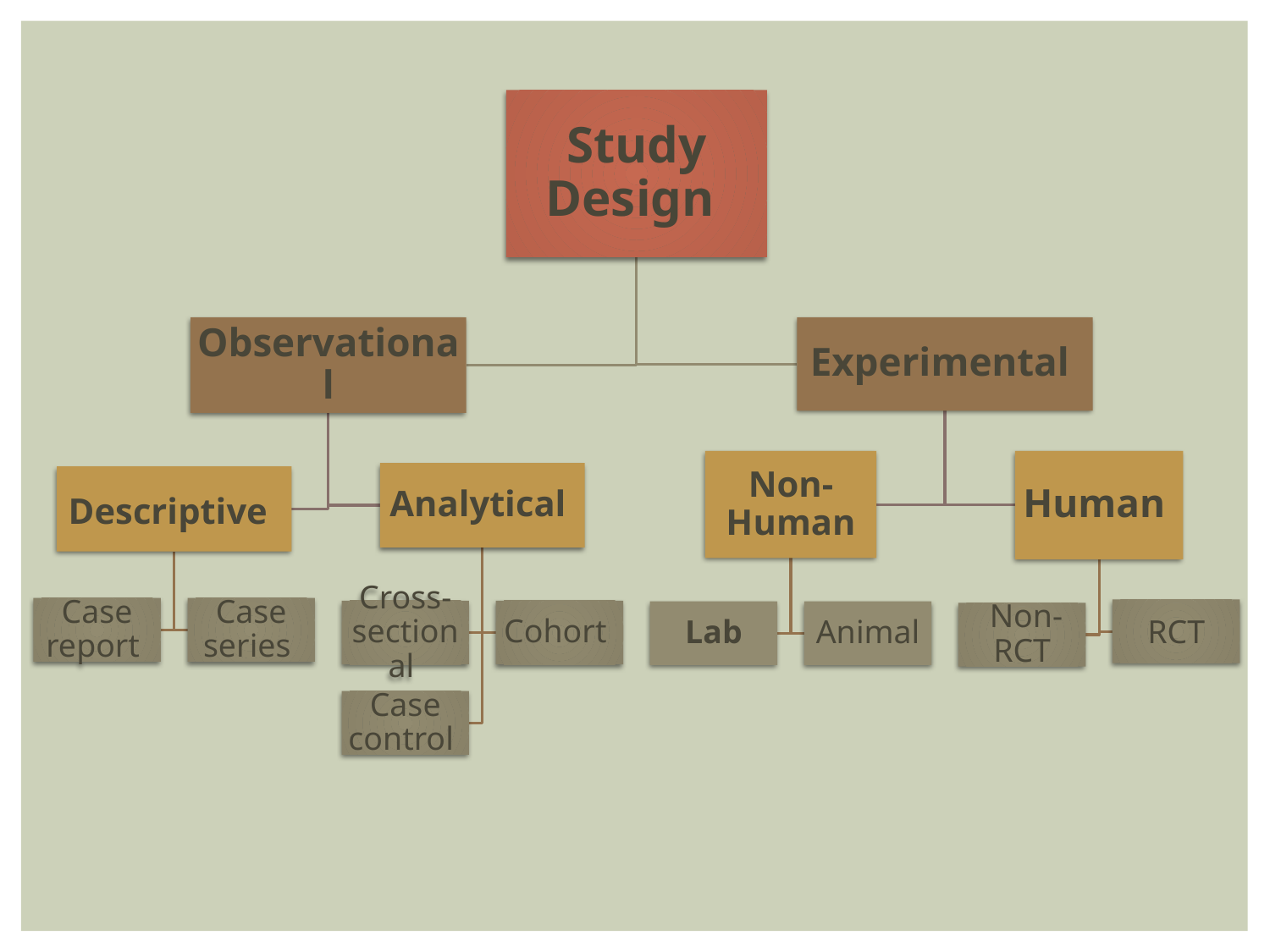

## Slide 13
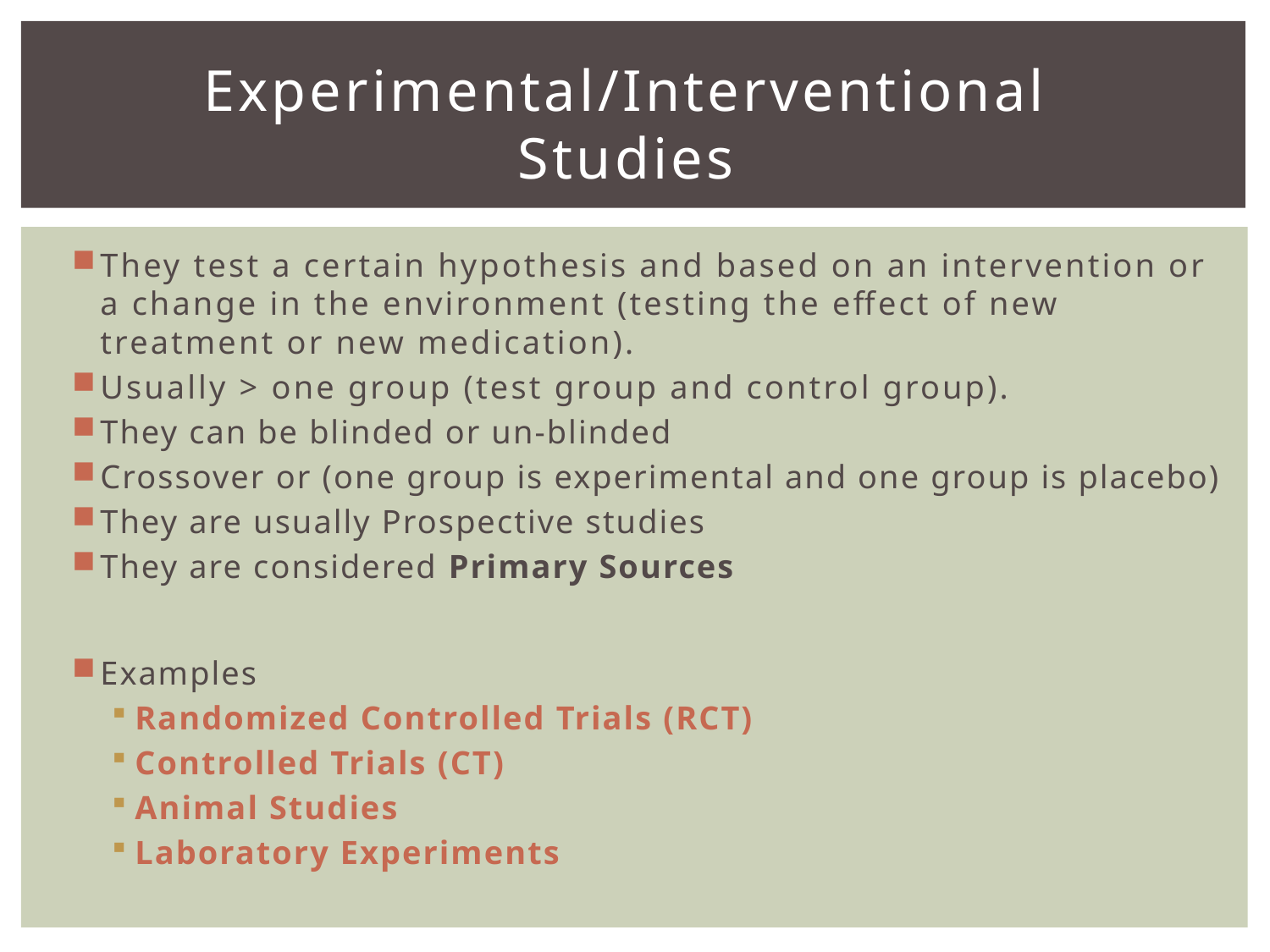

# Experimental/interventional Studies
They test a certain hypothesis and based on an intervention or a change in the environment (testing the effect of new treatment or new medication).
Usually > one group (test group and control group).
They can be blinded or un-blinded
Crossover or (one group is experimental and one group is placebo)
They are usually Prospective studies
They are considered Primary Sources
Examples
Randomized Controlled Trials (RCT)
Controlled Trials (CT)
Animal Studies
Laboratory Experiments

## Slide 14
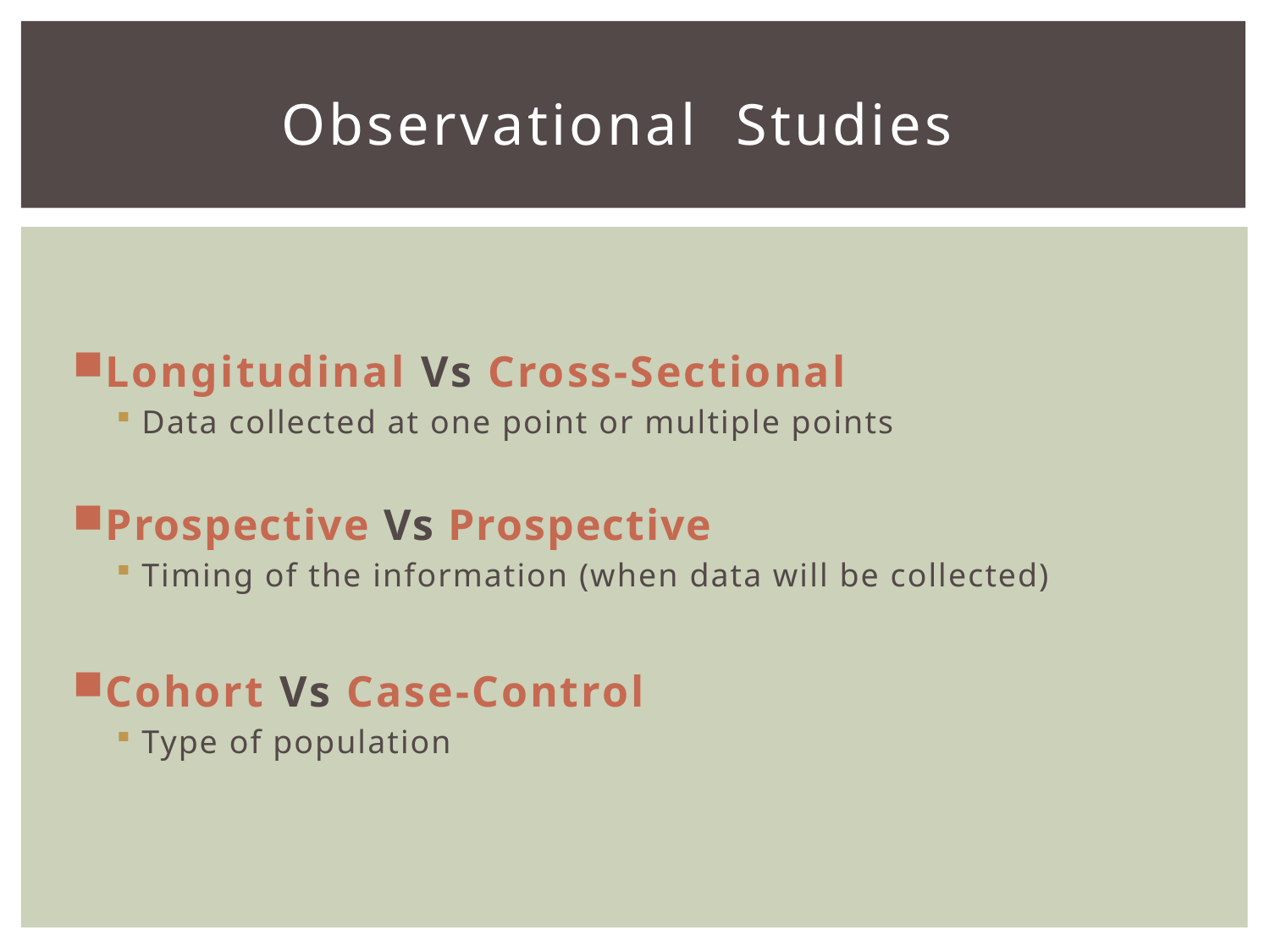

# Observational Studies
Longitudinal Vs Cross-Sectional
Data collected at one point or multiple points
Prospective Vs Prospective
Timing of the information (when data will be collected)
Cohort Vs Case-Control
Type of population

## Slide 15
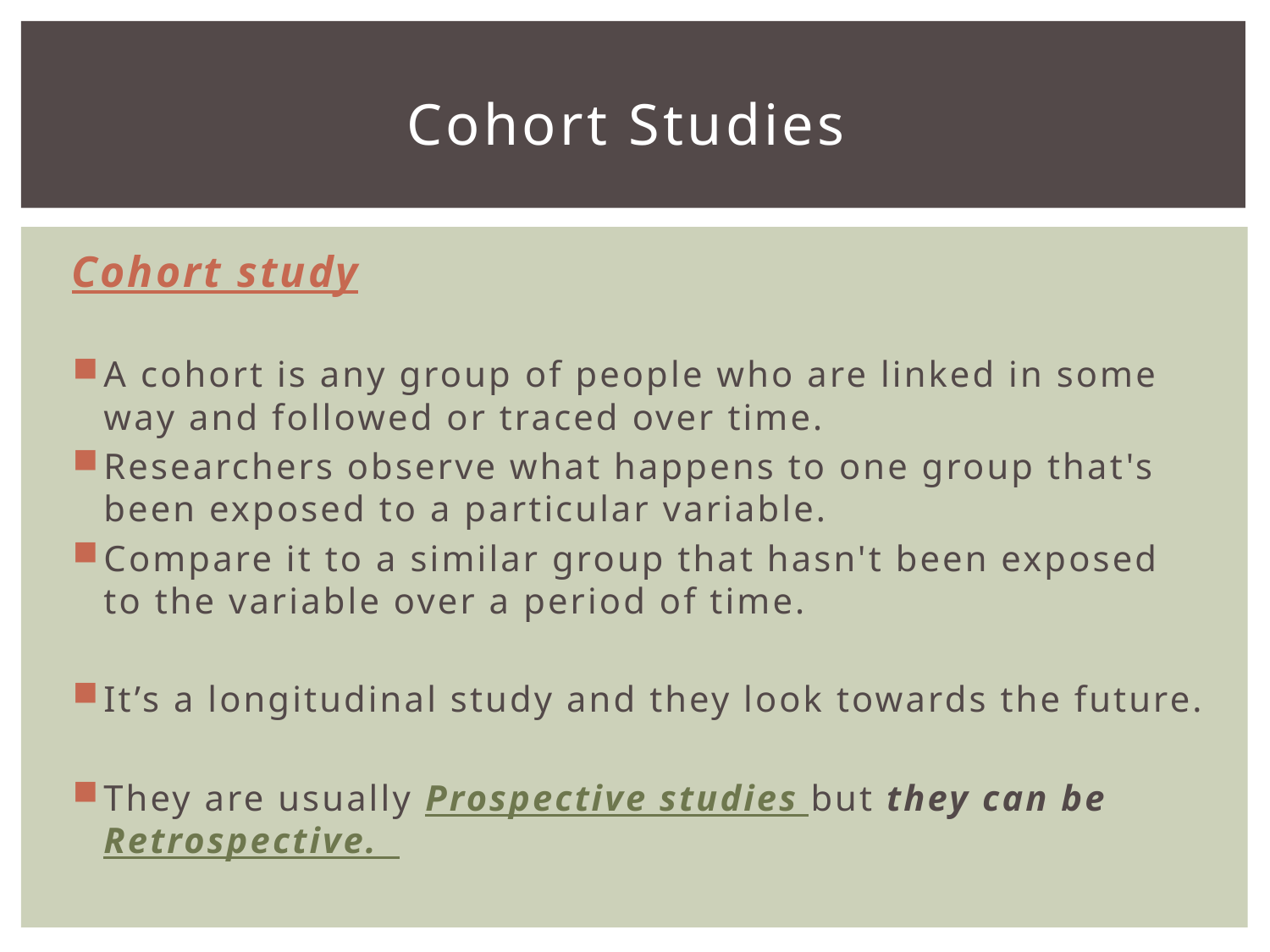

# Cohort Studies
Cohort study
A cohort is any group of people who are linked in some way and followed or traced over time.
Researchers observe what happens to one group that's been exposed to a particular variable.
Compare it to a similar group that hasn't been exposed to the variable over a period of time.
It’s a longitudinal study and they look towards the future.
They are usually Prospective studies but they can be Retrospective.

## Slide 16
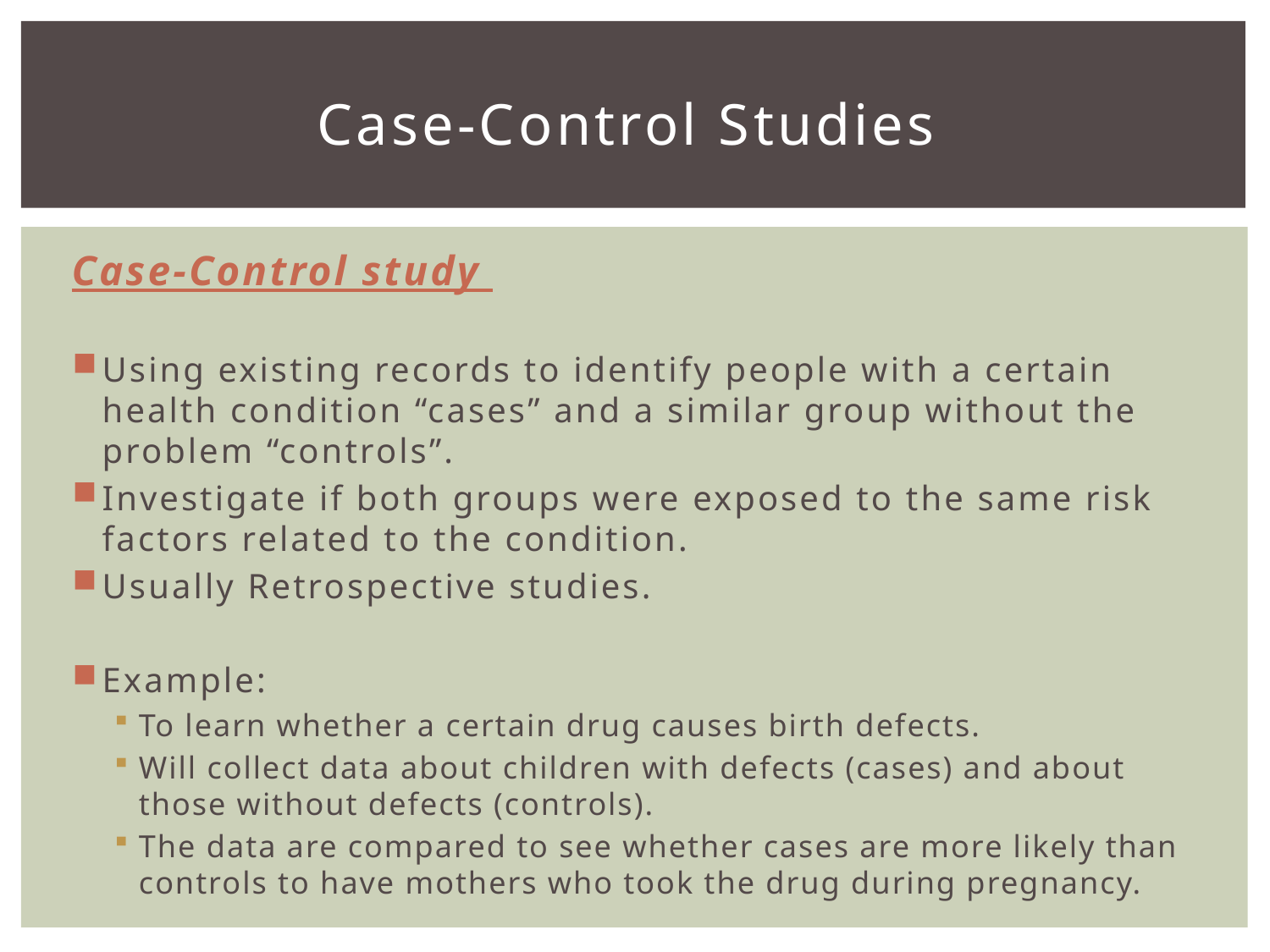

# Case-Control Studies
Case-Control study
Using existing records to identify people with a certain health condition “cases” and a similar group without the problem “controls”.
Investigate if both groups were exposed to the same risk factors related to the condition.
Usually Retrospective studies.
Example:
To learn whether a certain drug causes birth defects.
Will collect data about children with defects (cases) and about those without defects (controls).
The data are compared to see whether cases are more likely than controls to have mothers who took the drug during pregnancy.

## Slide 17
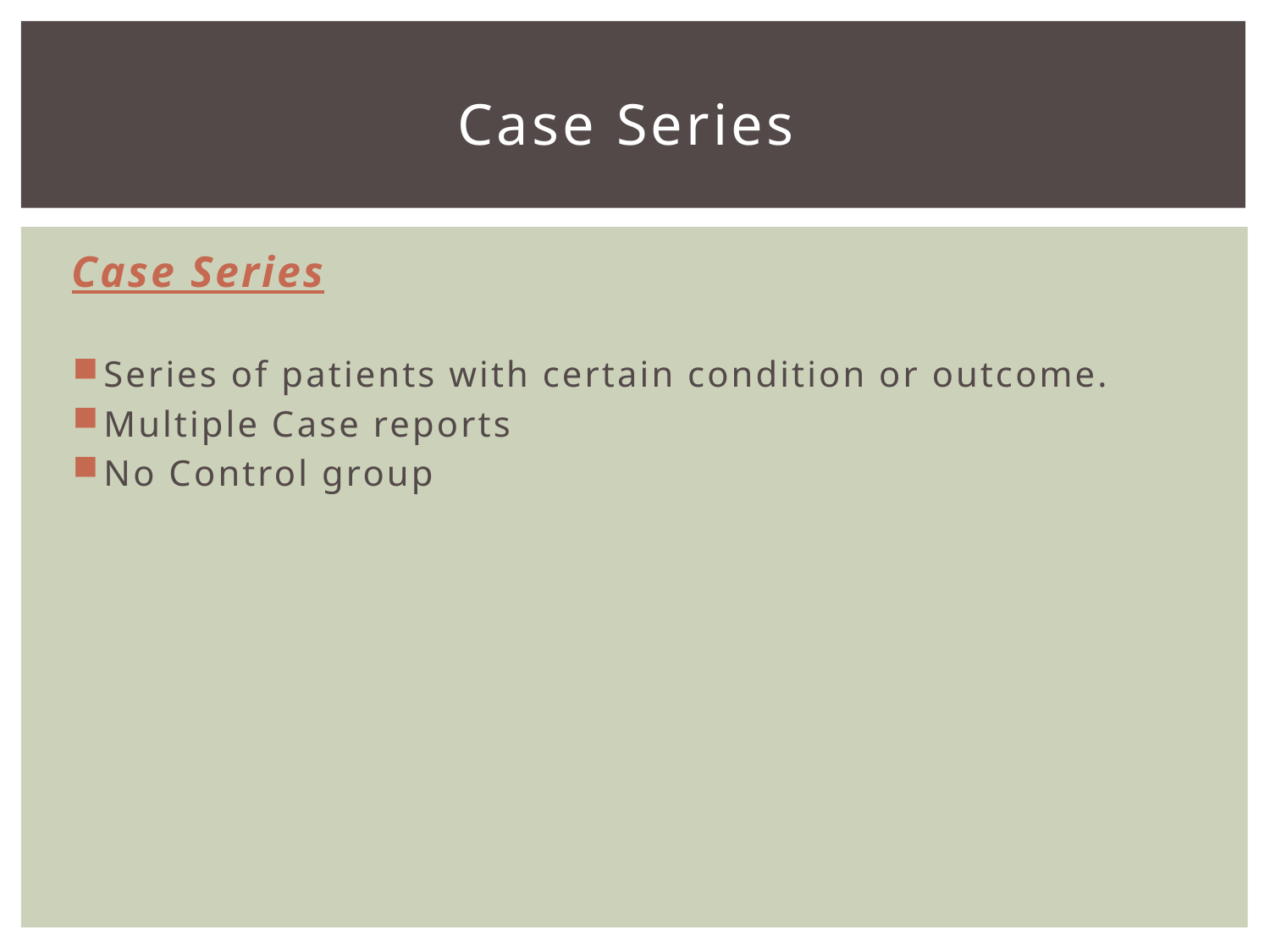

# Case series
Case Series
Series of patients with certain condition or outcome.
Multiple Case reports
No Control group

## Slide 18
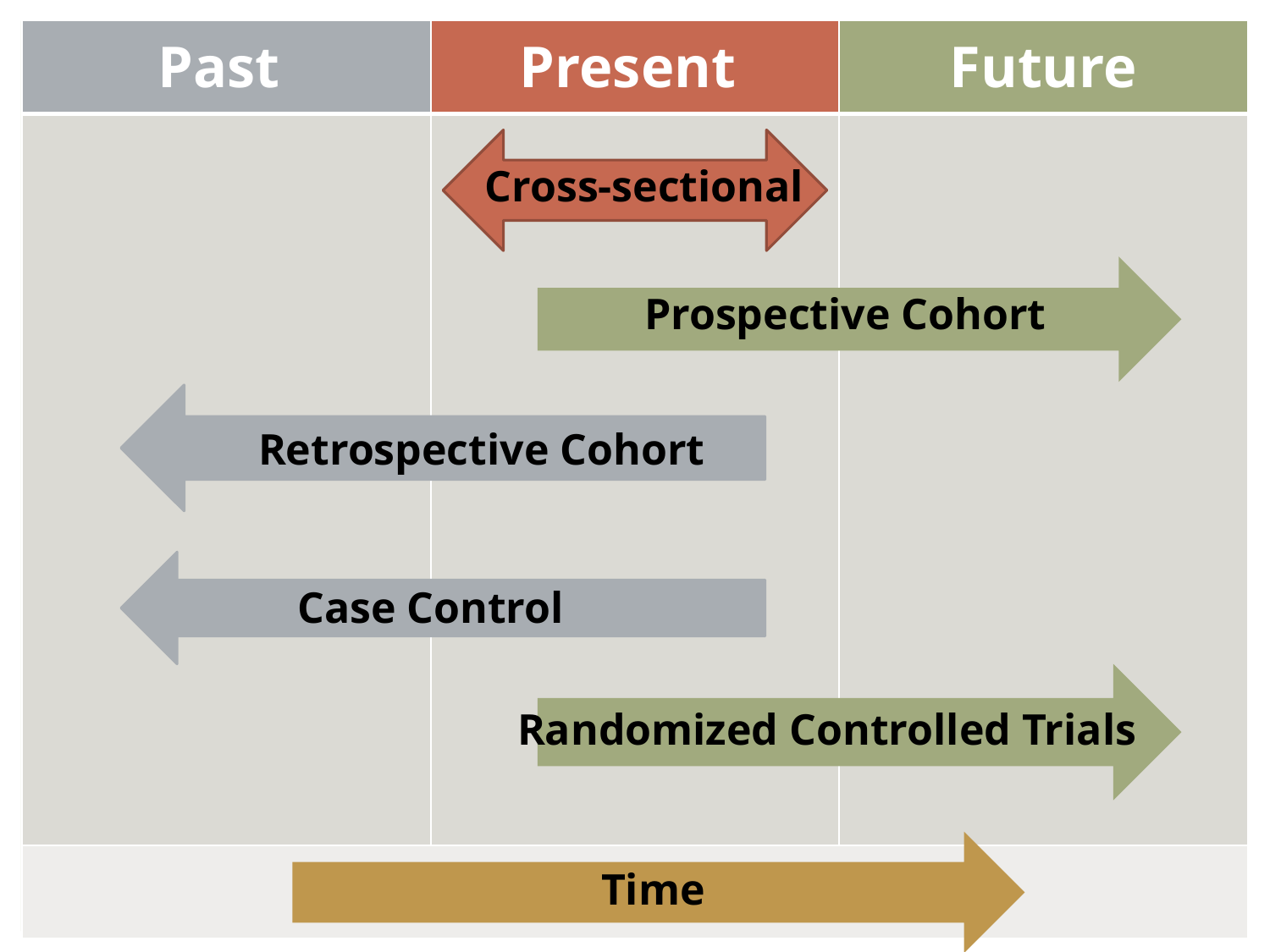

| Past | Present | Future |
| --- | --- | --- |
| | | |
| | | |
Cross-sectional
Prospective Cohort
Retrospective Cohort
Case Control
Randomized Controlled Trials
Time

## Slide 19
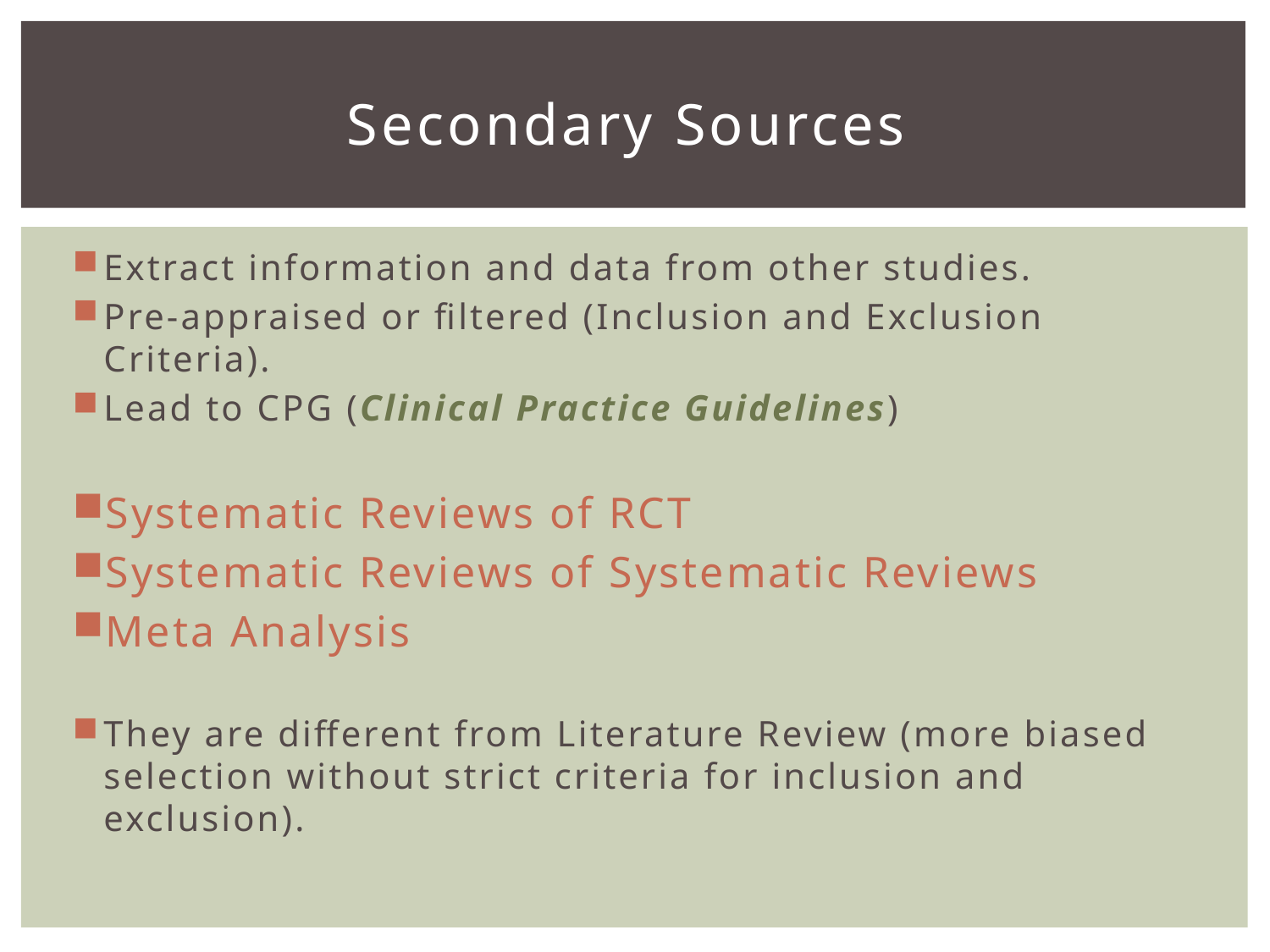

# Secondary Sources
Extract information and data from other studies.
Pre-appraised or filtered (Inclusion and Exclusion Criteria).
Lead to CPG (Clinical Practice Guidelines)
Systematic Reviews of RCT
Systematic Reviews of Systematic Reviews
Meta Analysis
They are different from Literature Review (more biased selection without strict criteria for inclusion and exclusion).

## Slide 20
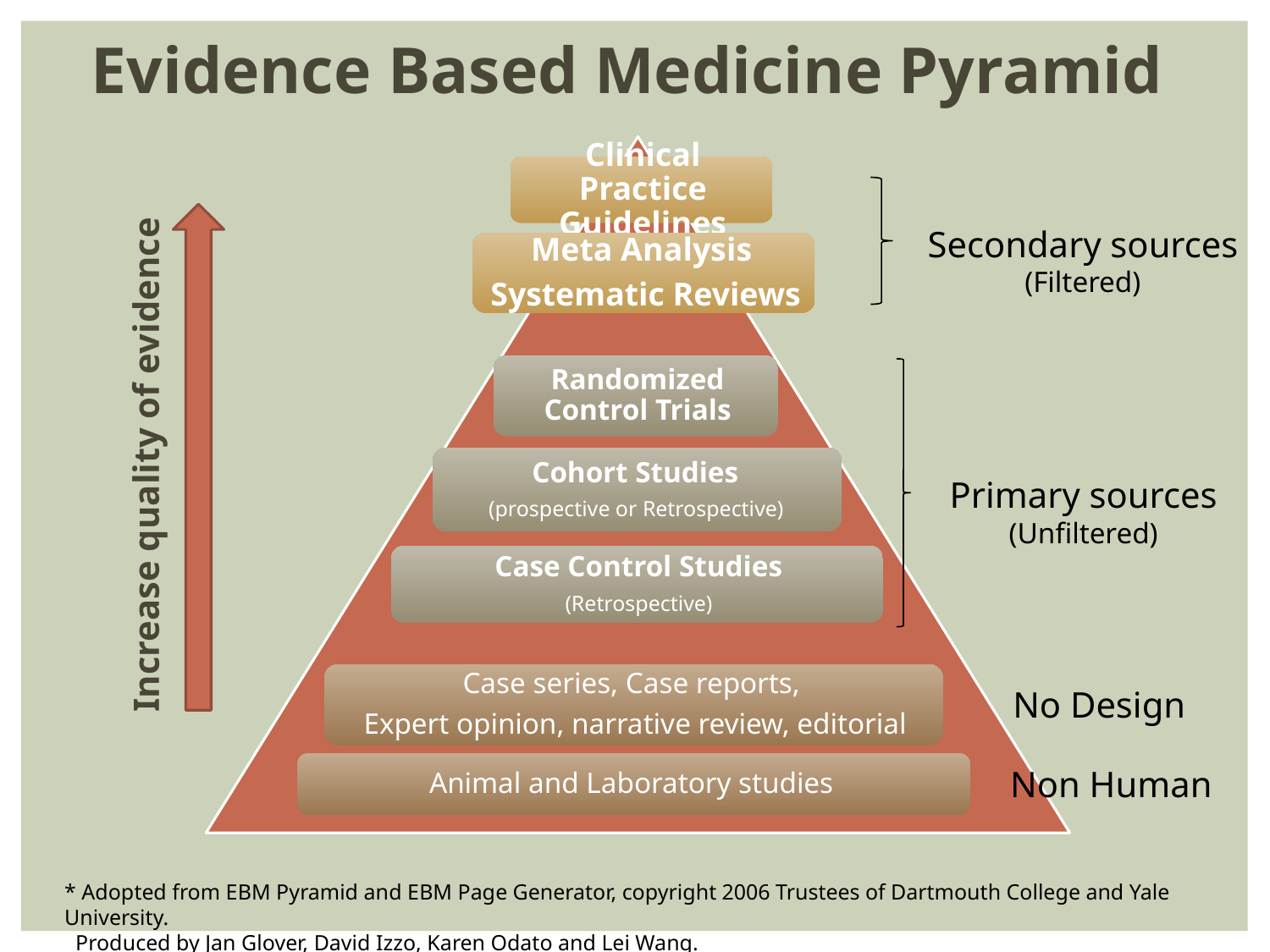

Evidence Based Medicine Pyramid
Secondary sources
(Filtered)
Increase quality of evidence
Primary sources
(Unfiltered)
No Design
Non Human
* Adopted from EBM Pyramid and EBM Page Generator, copyright 2006 Trustees of Dartmouth College and Yale University.
 Produced by Jan Glover, David Izzo, Karen Odato and Lei Wang.

## Slide 21
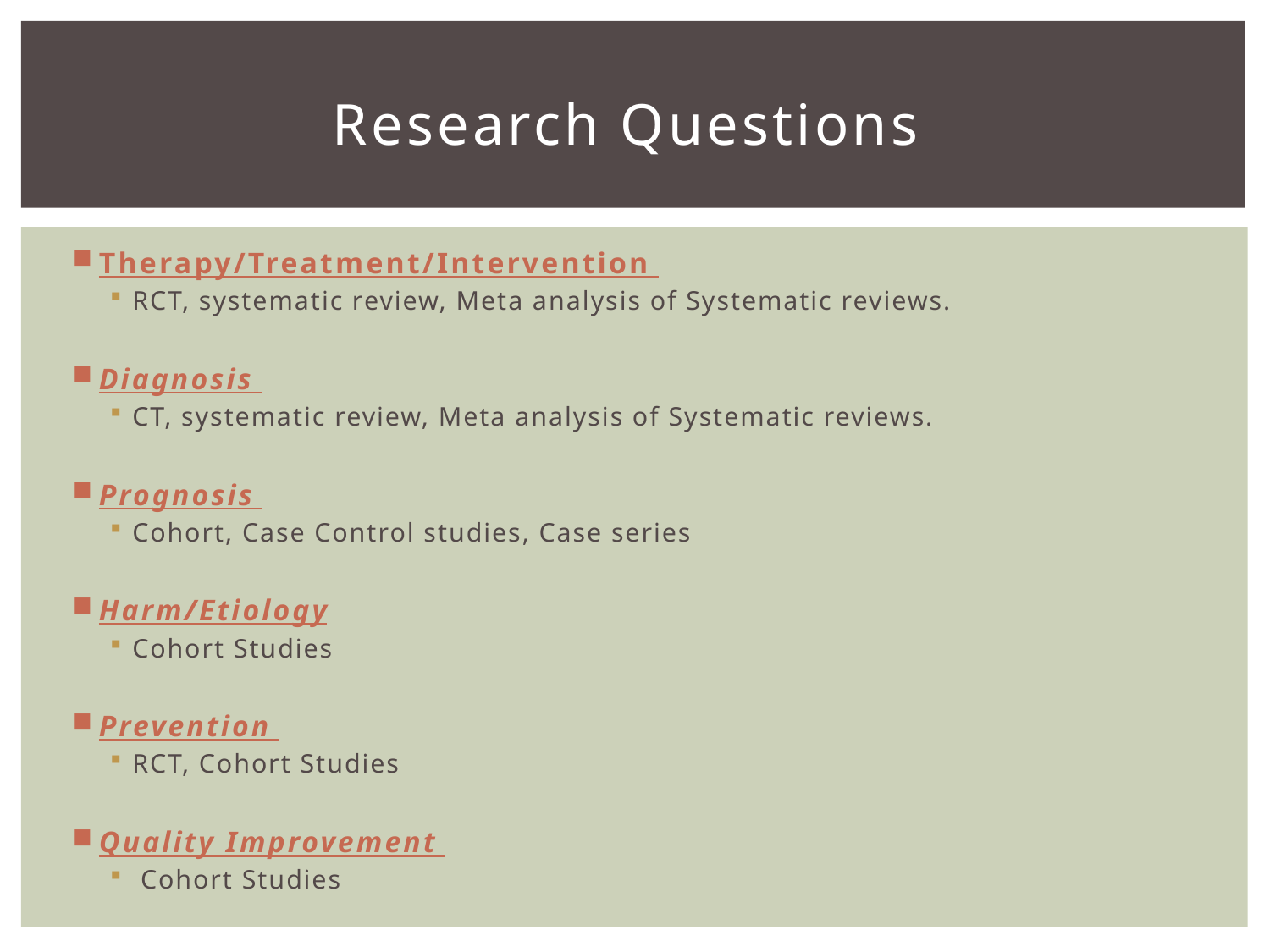

# Research Questions
Therapy/Treatment/Intervention
RCT, systematic review, Meta analysis of Systematic reviews.
Diagnosis
CT, systematic review, Meta analysis of Systematic reviews.
Prognosis
Cohort, Case Control studies, Case series
Harm/Etiology
Cohort Studies
Prevention
RCT, Cohort Studies
Quality Improvement
 Cohort Studies

## Slide 22
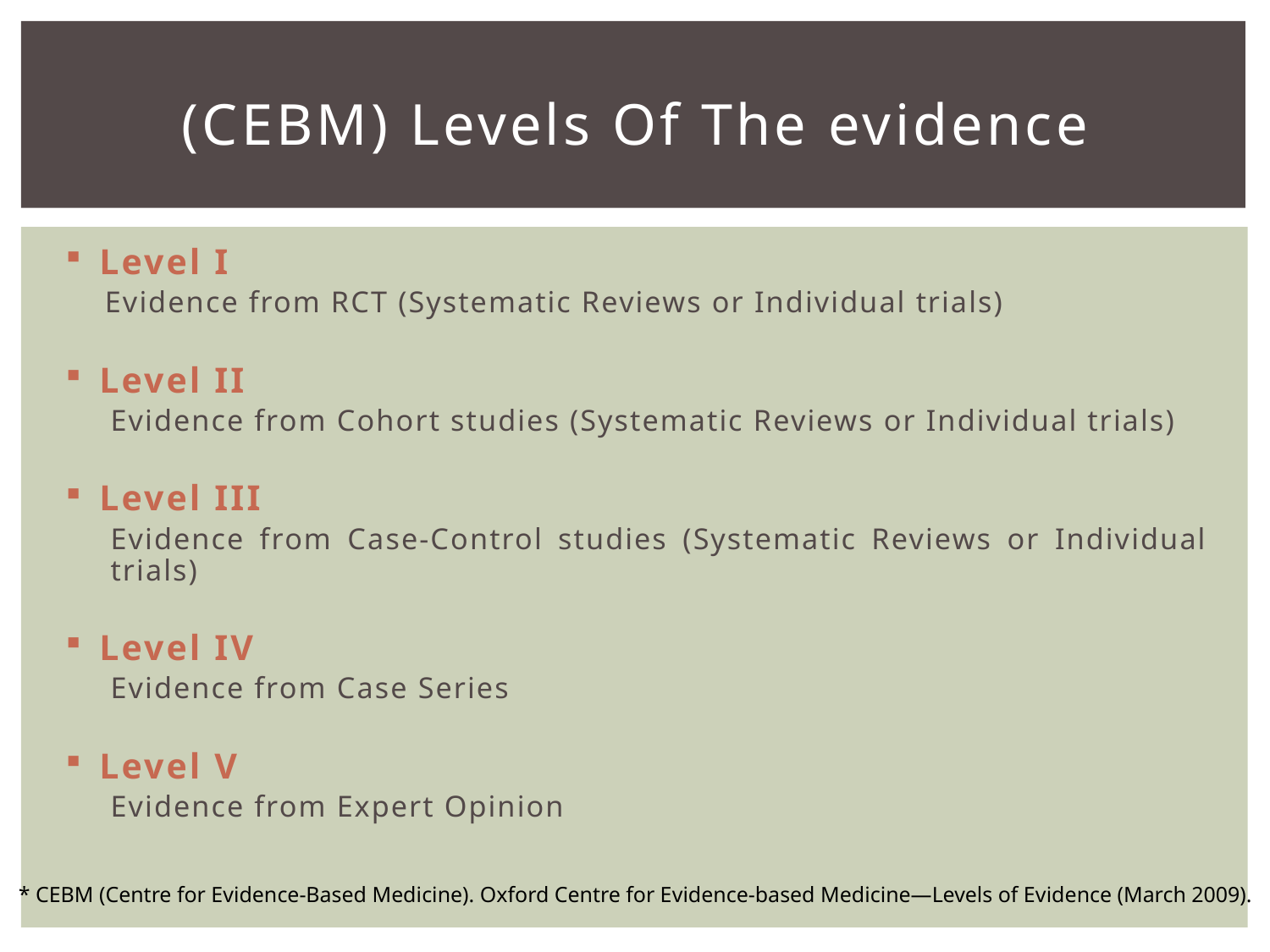

# (CEBM) Levels of the evidence
Level I
Evidence from RCT (Systematic Reviews or Individual trials)
Level II
Evidence from Cohort studies (Systematic Reviews or Individual trials)
Level III
Evidence from Case-Control studies (Systematic Reviews or Individual trials)
Level IV
Evidence from Case Series
Level V
Evidence from Expert Opinion
* CEBM (Centre for Evidence-Based Medicine). Oxford Centre for Evidence-based Medicine—Levels of Evidence (March 2009).

## Slide 23
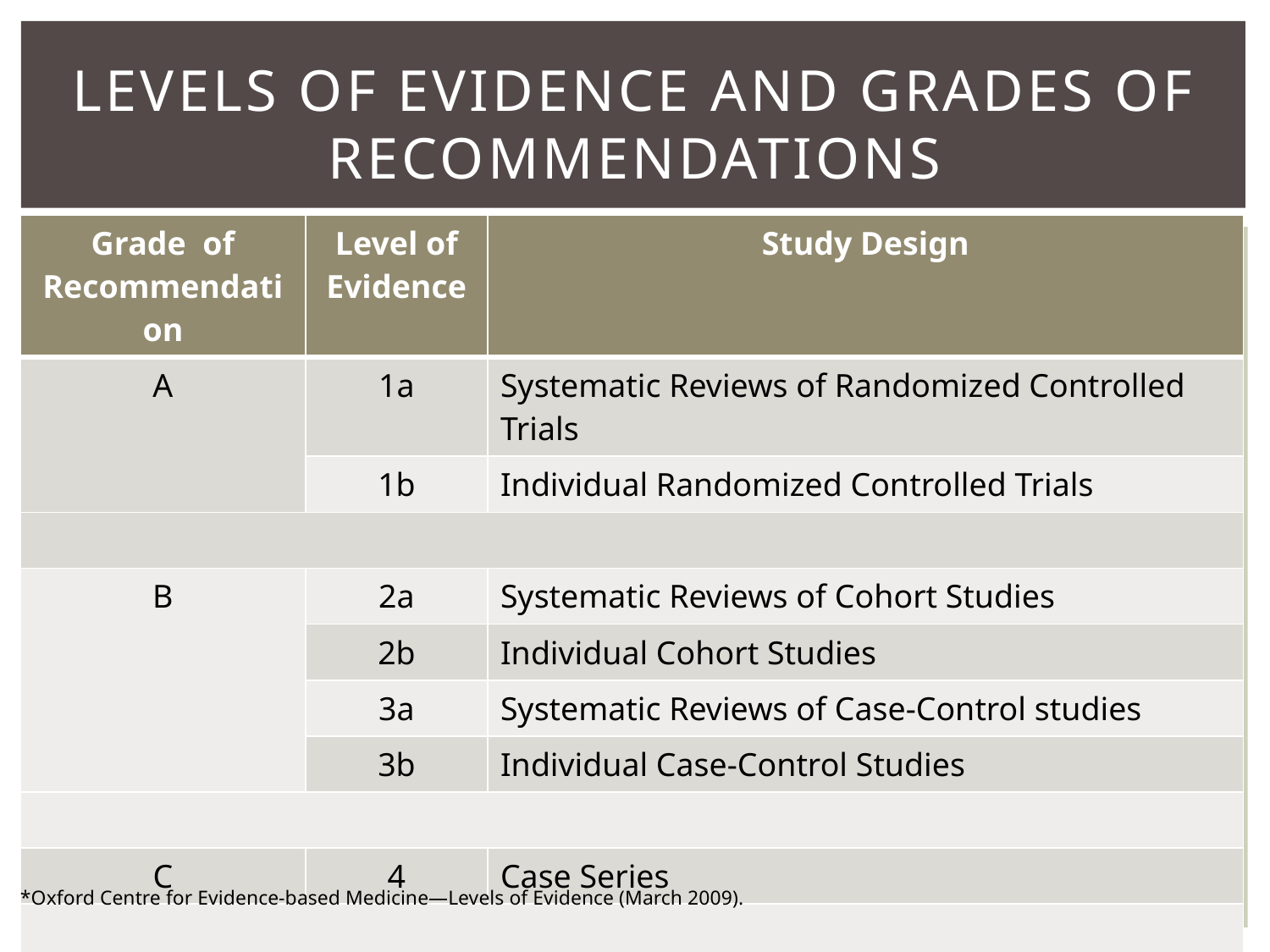

# Levels of Evidence and Grades of Recommendations
| Grade of Recommendation | Level of Evidence | Study Design |
| --- | --- | --- |
| A | 1a | Systematic Reviews of Randomized Controlled Trials |
| | 1b | Individual Randomized Controlled Trials |
| | | |
| B | 2a | Systematic Reviews of Cohort Studies |
| | 2b | Individual Cohort Studies |
| | 3a | Systematic Reviews of Case-Control studies |
| | 3b | Individual Case-Control Studies |
| | | |
| C | 4 | Case Series |
| | | |
| D | 5 | Expert Opinion without critical appraisal |
*Oxford Centre for Evidence-based Medicine—Levels of Evidence (March 2009).

## Slide 24
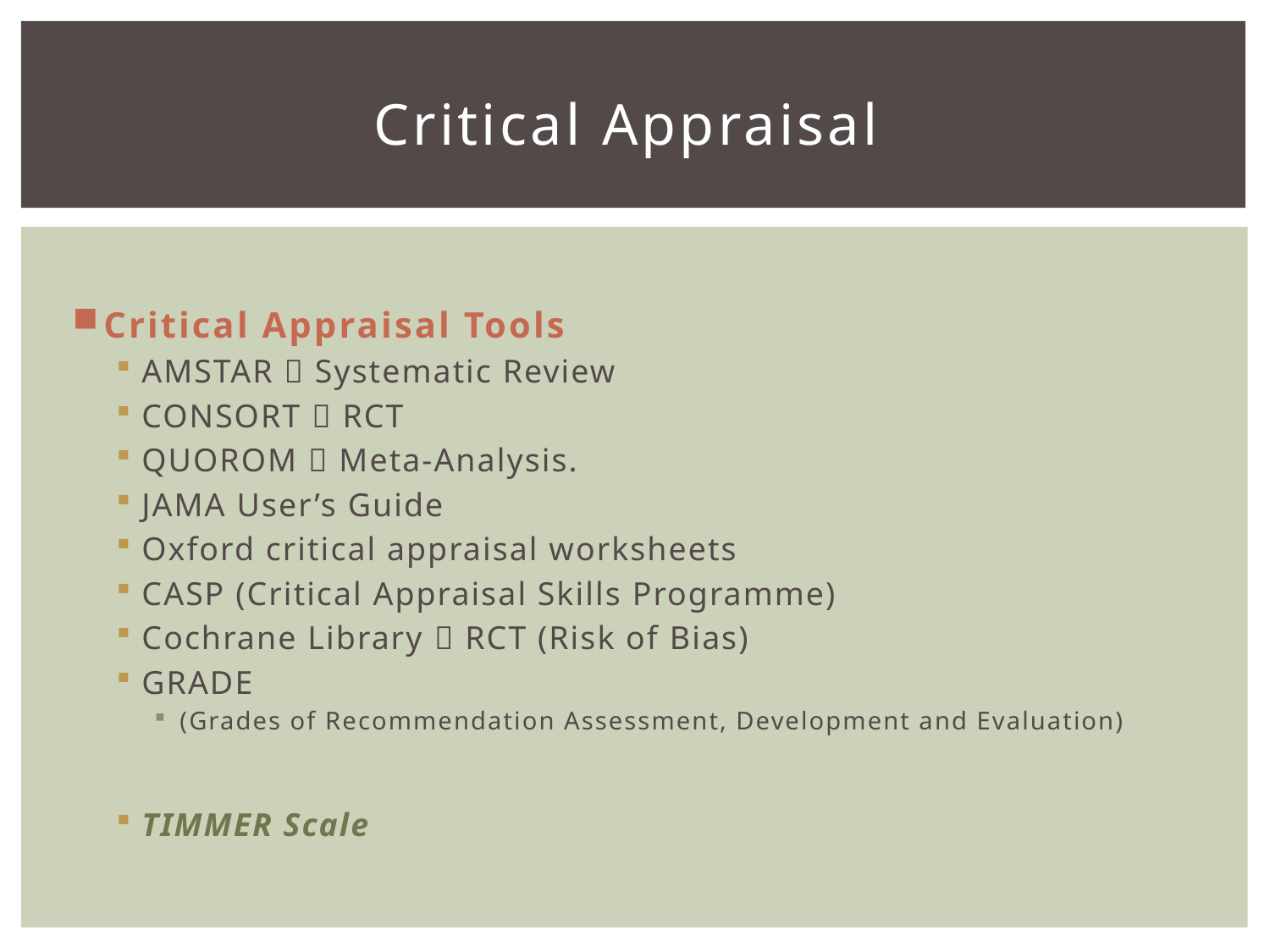

# Critical Appraisal
Critical Appraisal Tools
AMSTAR  Systematic Review
CONSORT  RCT
QUOROM  Meta-Analysis.
JAMA User’s Guide
Oxford critical appraisal worksheets
CASP (Critical Appraisal Skills Programme)
Cochrane Library  RCT (Risk of Bias)
GRADE
(Grades of Recommendation Assessment, Development and Evaluation)
TIMMER Scale

## Slide 25
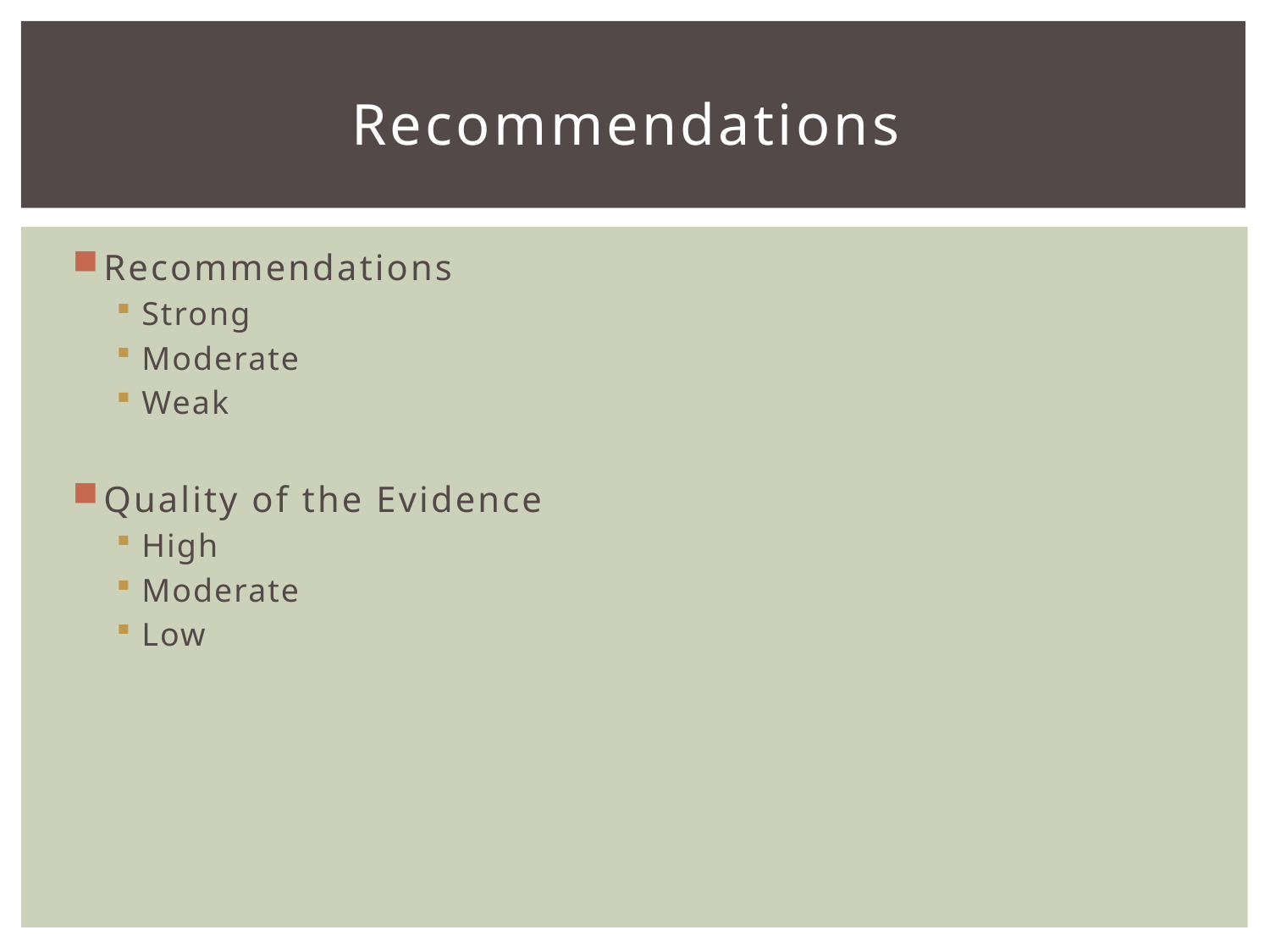

# Recommendations
Recommendations
Strong
Moderate
Weak
Quality of the Evidence
High
Moderate
Low

## Slide 26
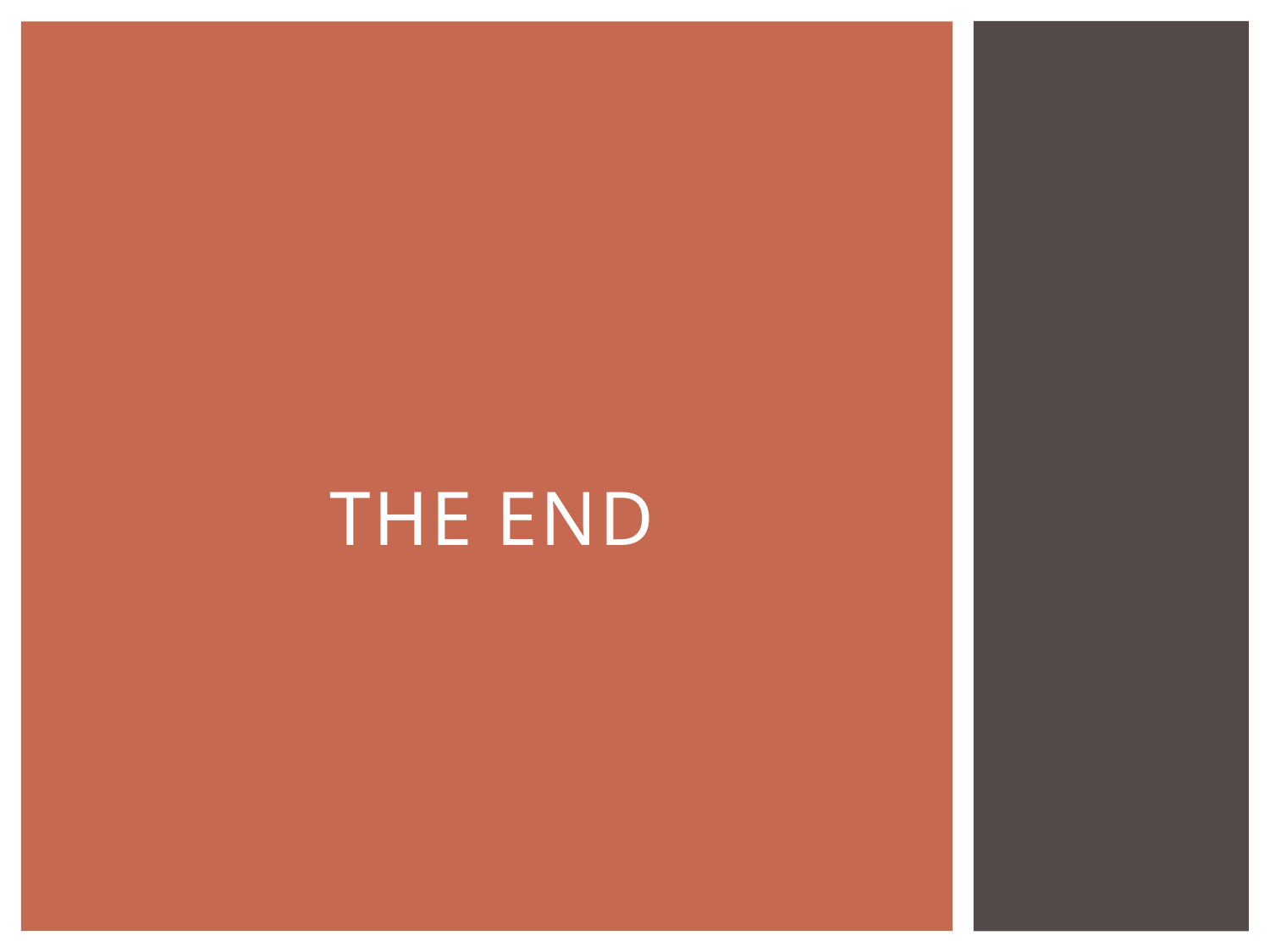

# The End
