# Supplementary material for: Using the Timmer Scale to Standardize Pediatric Dentistry Residents’ Scientific Appraisal Skills
Source: MedEdPORTAL. 2021 Feb 12;17:11101. doi: 10.15766/mep_2374-8265.11101 (PMC7880256; doi:10.15766/mep_2374-8265.11101)
Supplement: Supplementary file 1 — Introductory Course Material (EBP).pptxJournal Club Course Introduction.pptxQuality Assessment Score Sheet.docxStudy Design and Total Possible Points Form.docxArticles Evaluation Form.docxCourse Evaluation Form.docxPreclass and Remediation Reading Assignments.docx [file mep_2374-8265.11101-s001.zip › B. Journal Club Course Introduction.pptx]

## Slide 1
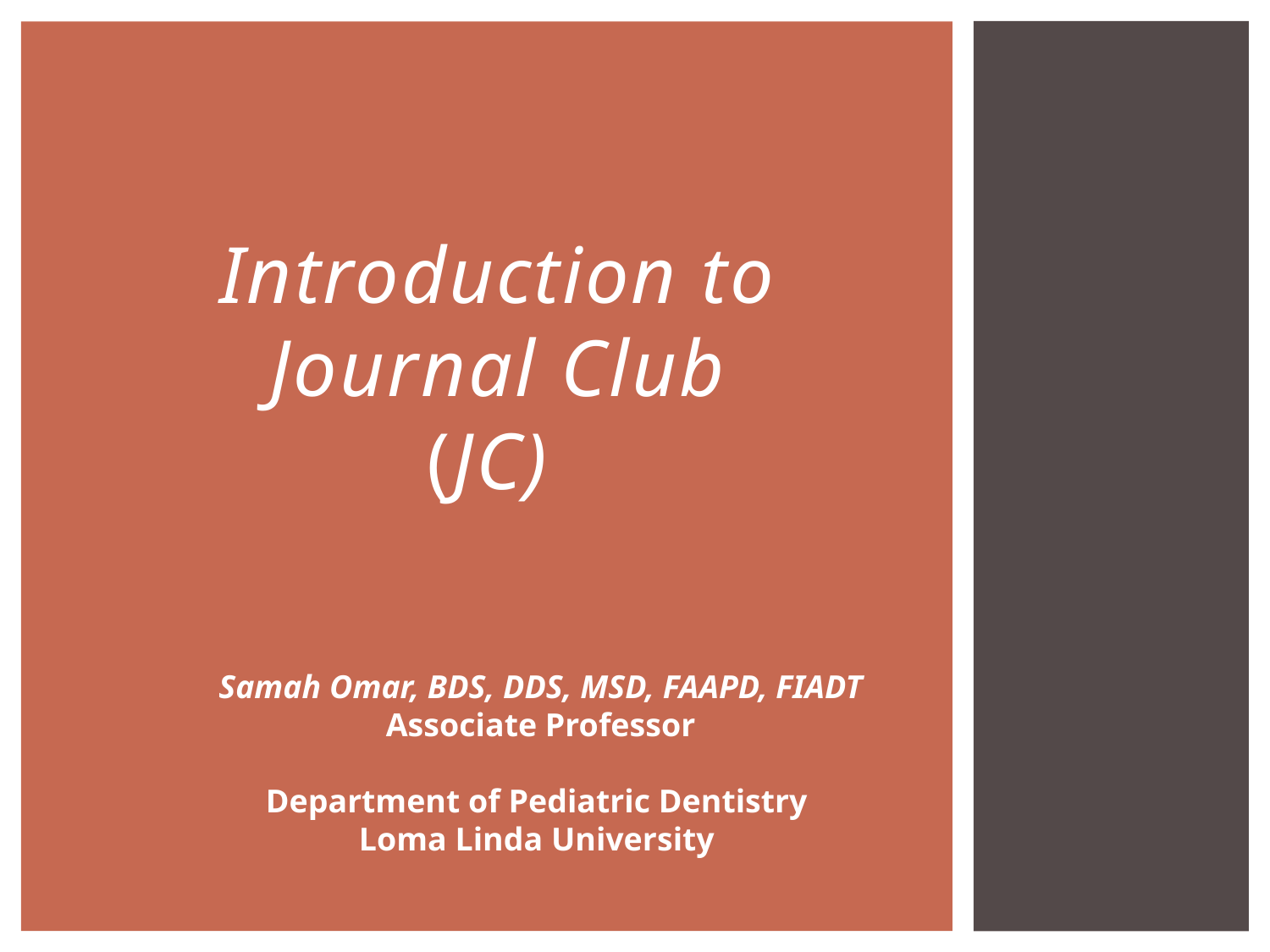

# Introduction to Journal Club (JC)
Samah Omar, BDS, DDS, MSD, FAAPD, FIADT
Associate Professor
Department of Pediatric Dentistry
Loma Linda University

## Slide 2
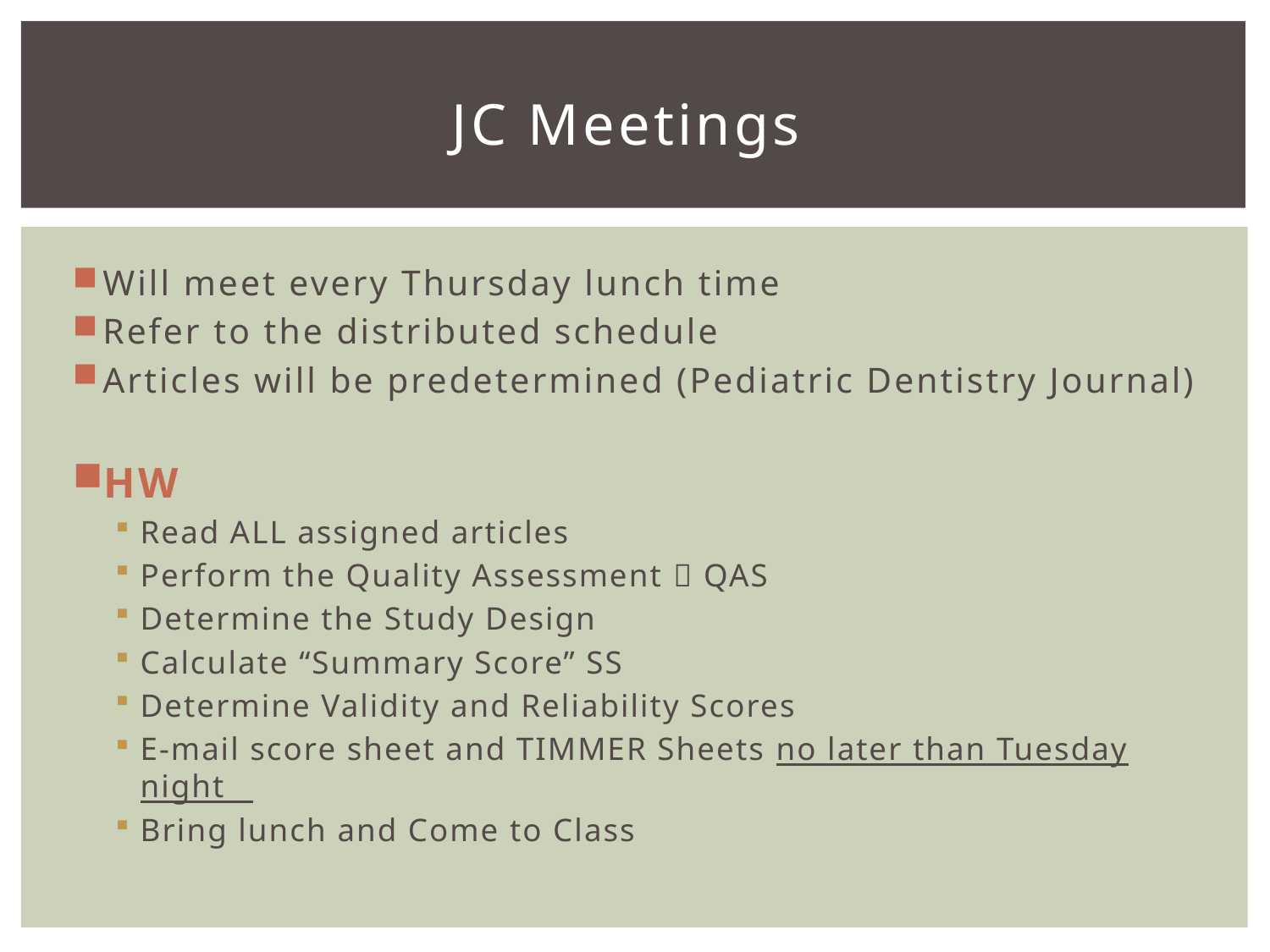

# JC Meetings
Will meet every Thursday lunch time
Refer to the distributed schedule
Articles will be predetermined (Pediatric Dentistry Journal)
HW
Read ALL assigned articles
Perform the Quality Assessment  QAS
Determine the Study Design
Calculate “Summary Score” SS
Determine Validity and Reliability Scores
E-mail score sheet and TIMMER Sheets no later than Tuesday night
Bring lunch and Come to Class

## Slide 3
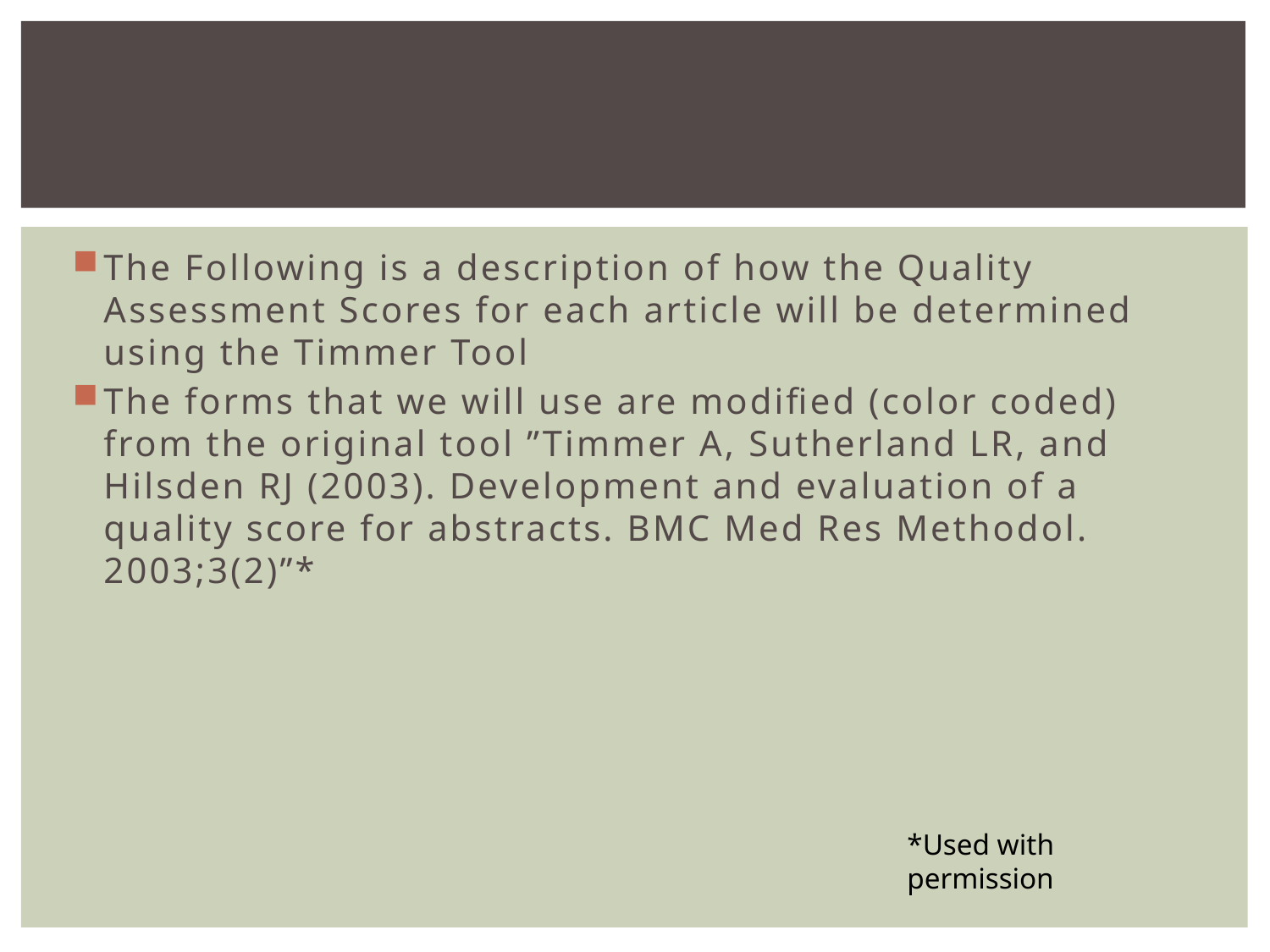

#
The Following is a description of how the Quality Assessment Scores for each article will be determined using the Timmer Tool
The forms that we will use are modified (color coded) from the original tool ”Timmer A, Sutherland LR, and Hilsden RJ (2003). Development and evaluation of a quality score for abstracts. BMC Med Res Methodol. 2003;3(2)”*
*Used with permission

## Slide 4
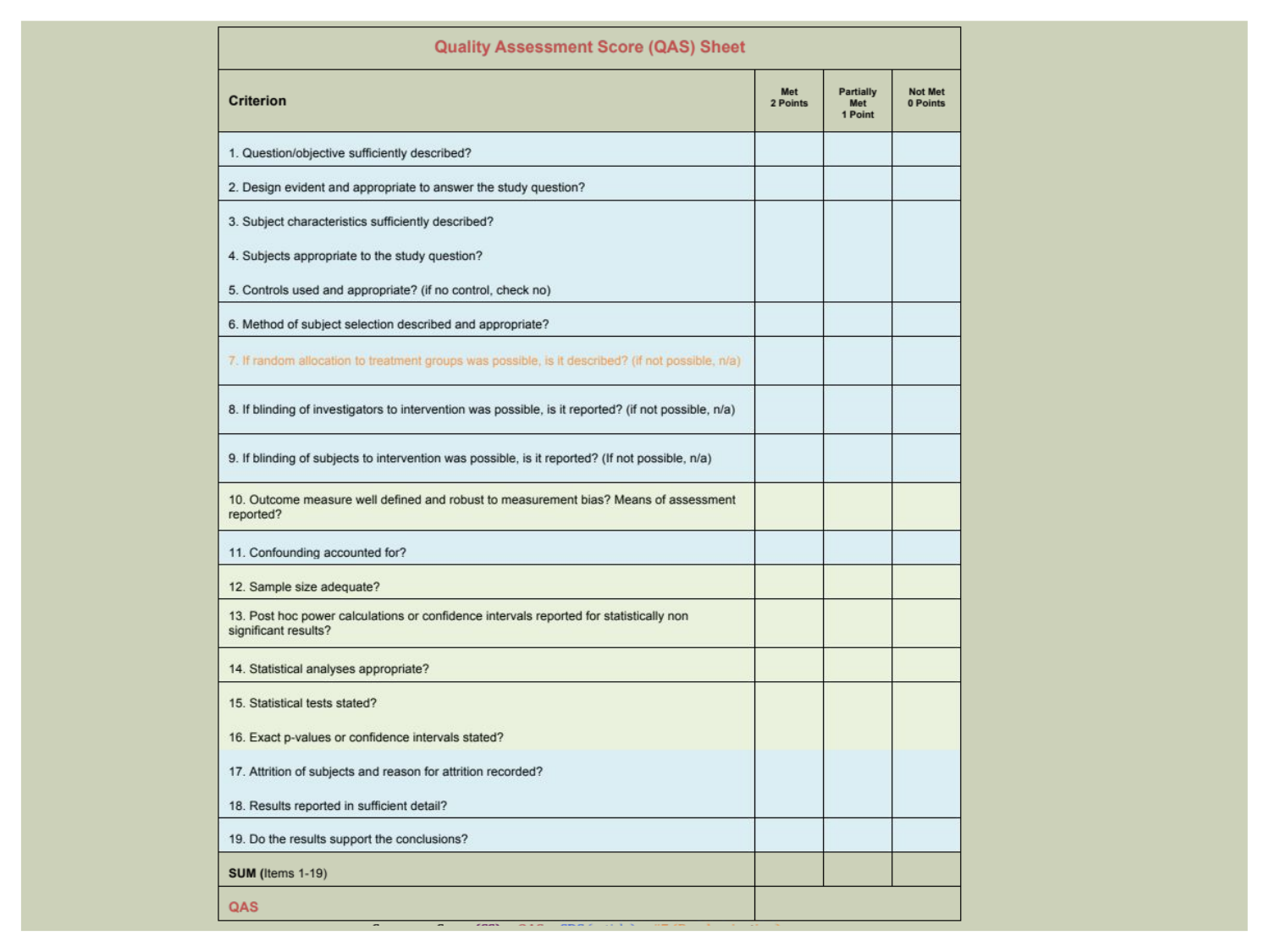

## Slide 5
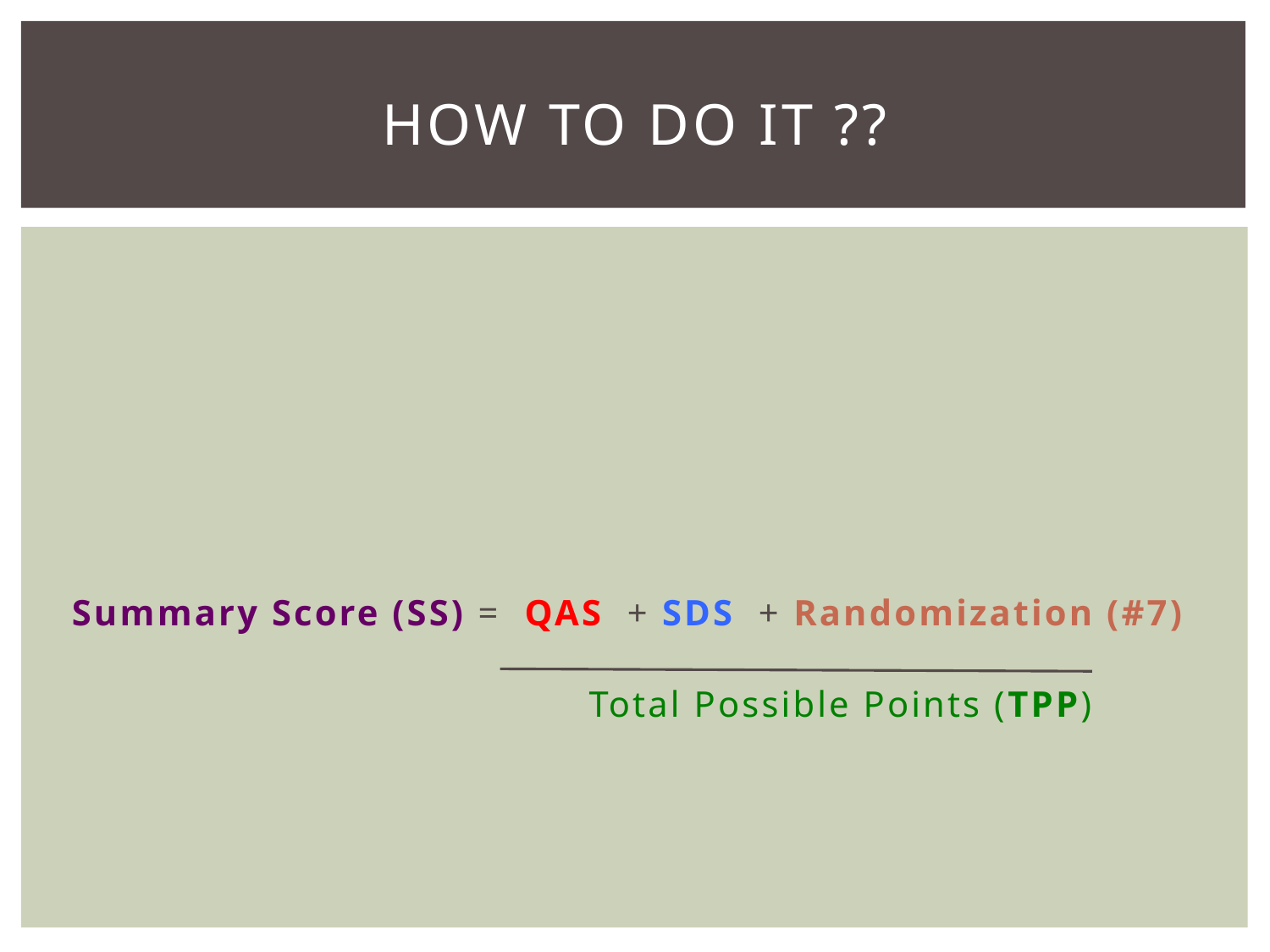

# How to Do it ??
Summary Score (SS) = QAS + SDS + Randomization (#7)
 Total Possible Points (TPP)

## Slide 6
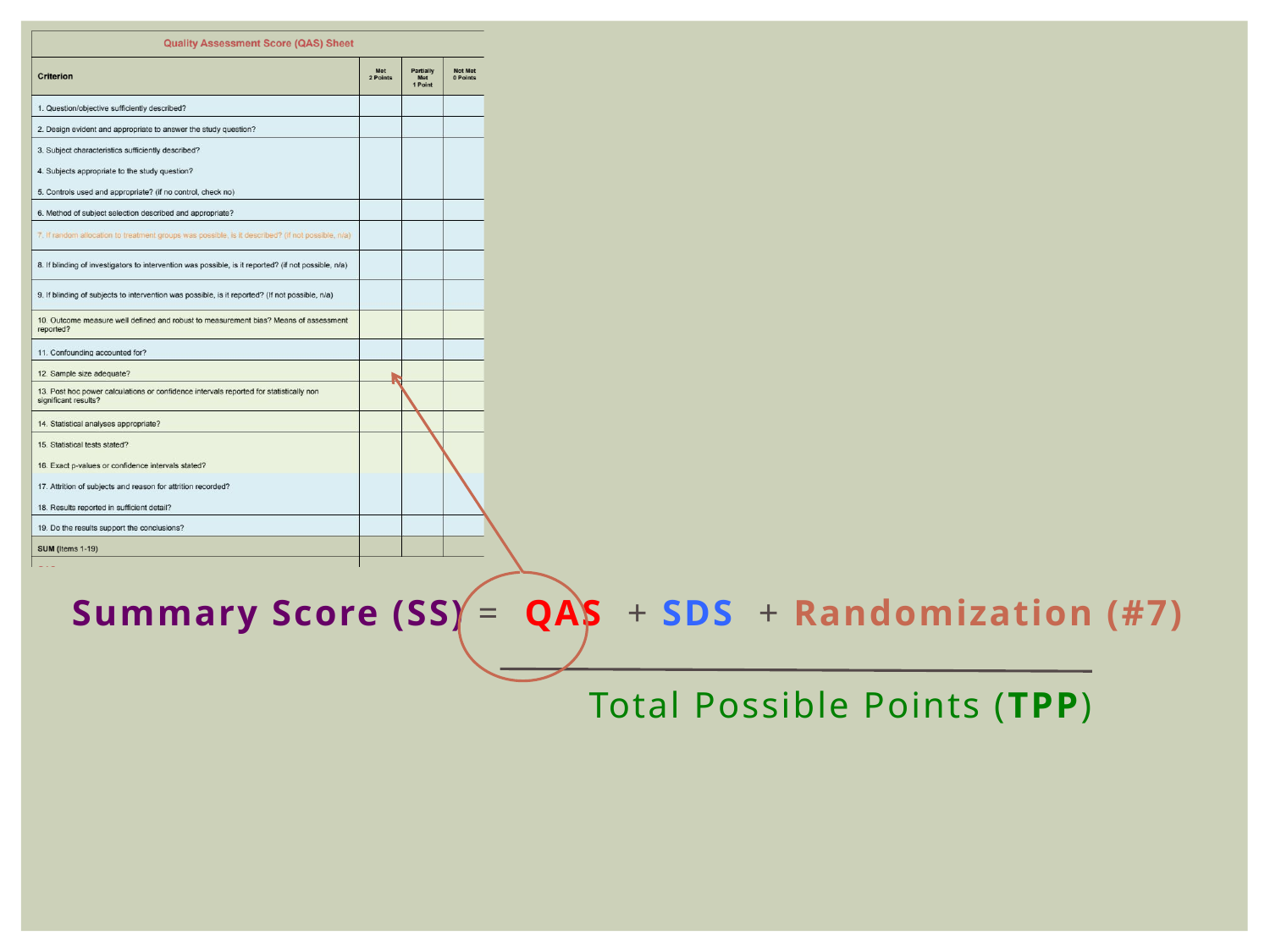

Summary Score (SS) = QAS + SDS + Randomization (#7)
 Total Possible Points (TPP)

## Slide 7
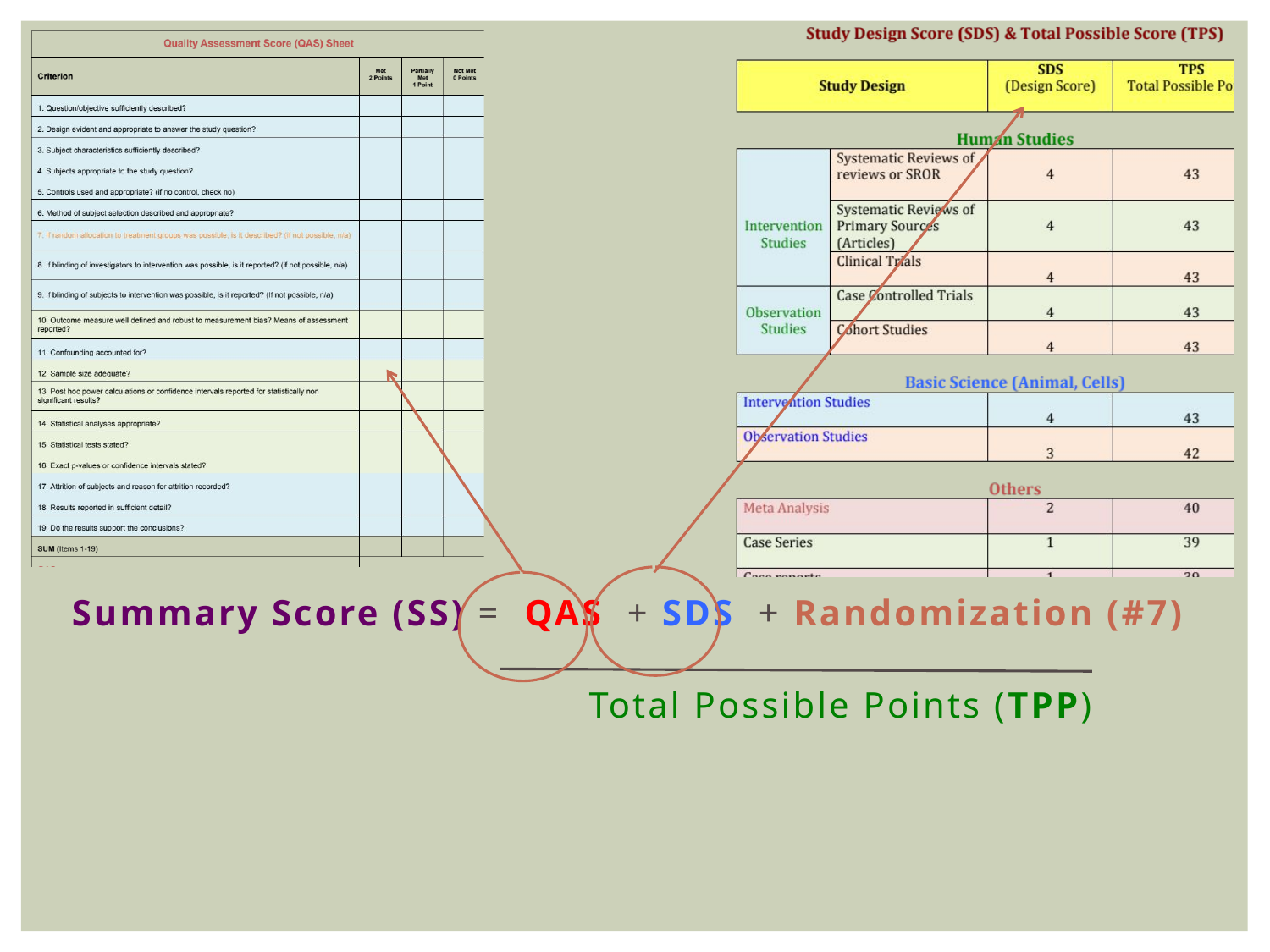

Summary Score (SS) = QAS + SDS + Randomization (#7)
 Total Possible Points (TPP)

## Slide 8
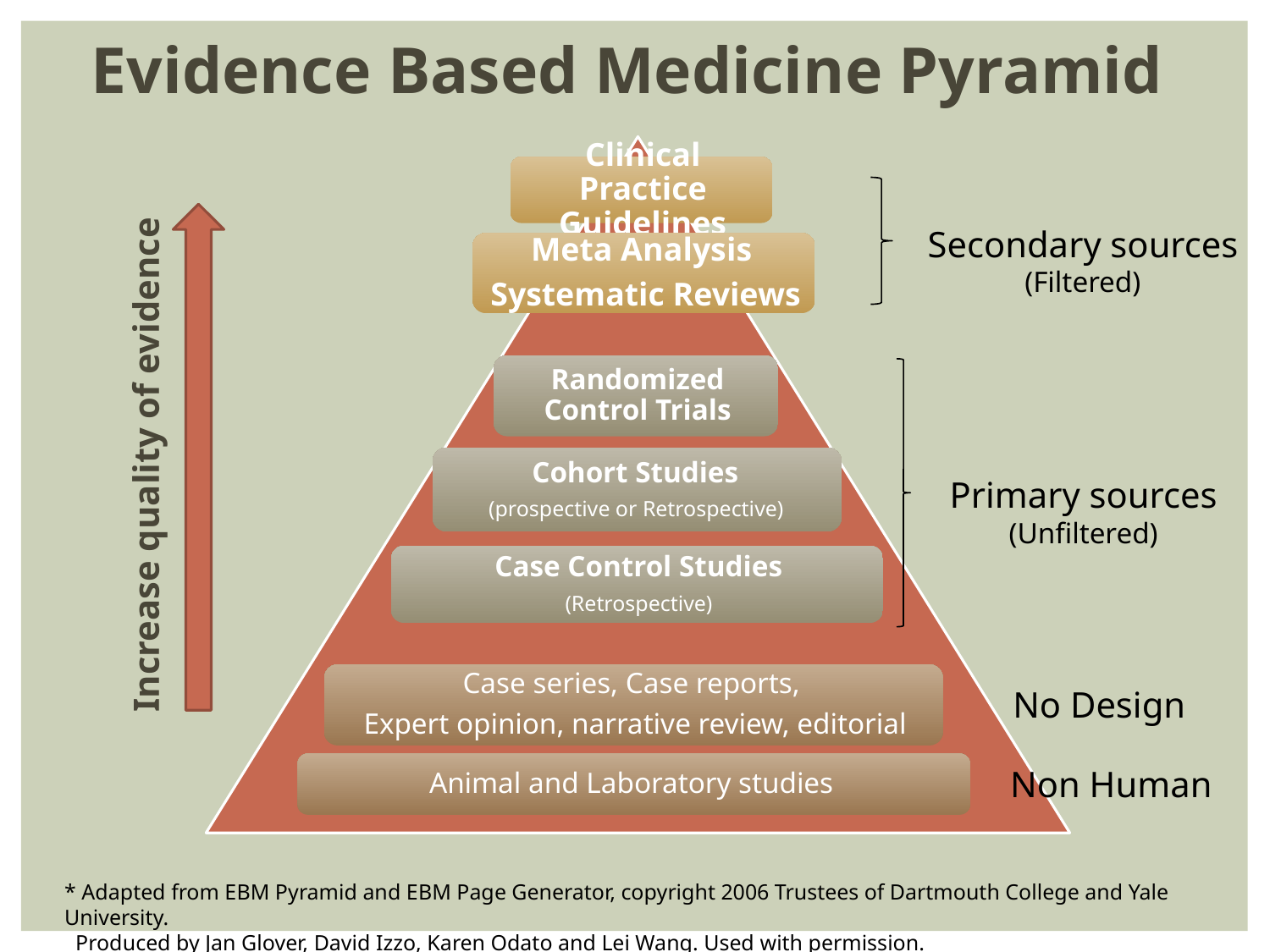

Evidence Based Medicine Pyramid
Secondary sources
(Filtered)
Increase quality of evidence
Primary sources
(Unfiltered)
No Design
Non Human
* Adapted from EBM Pyramid and EBM Page Generator, copyright 2006 Trustees of Dartmouth College and Yale University.
 Produced by Jan Glover, David Izzo, Karen Odato and Lei Wang. Used with permission.

## Slide 9
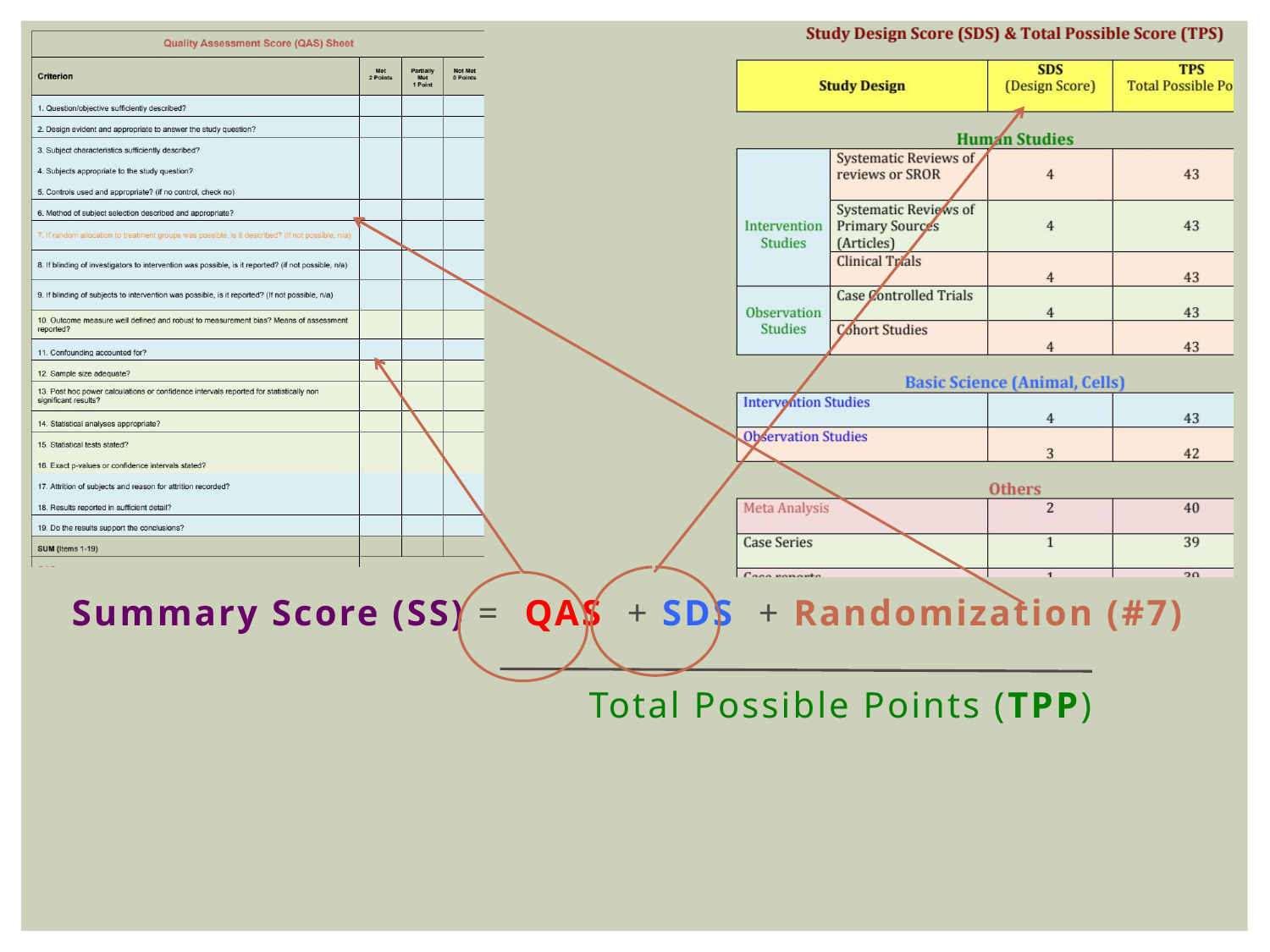

Summary Score (SS) = QAS + SDS + Randomization (#7)
 Total Possible Points (TPP)

## Slide 10
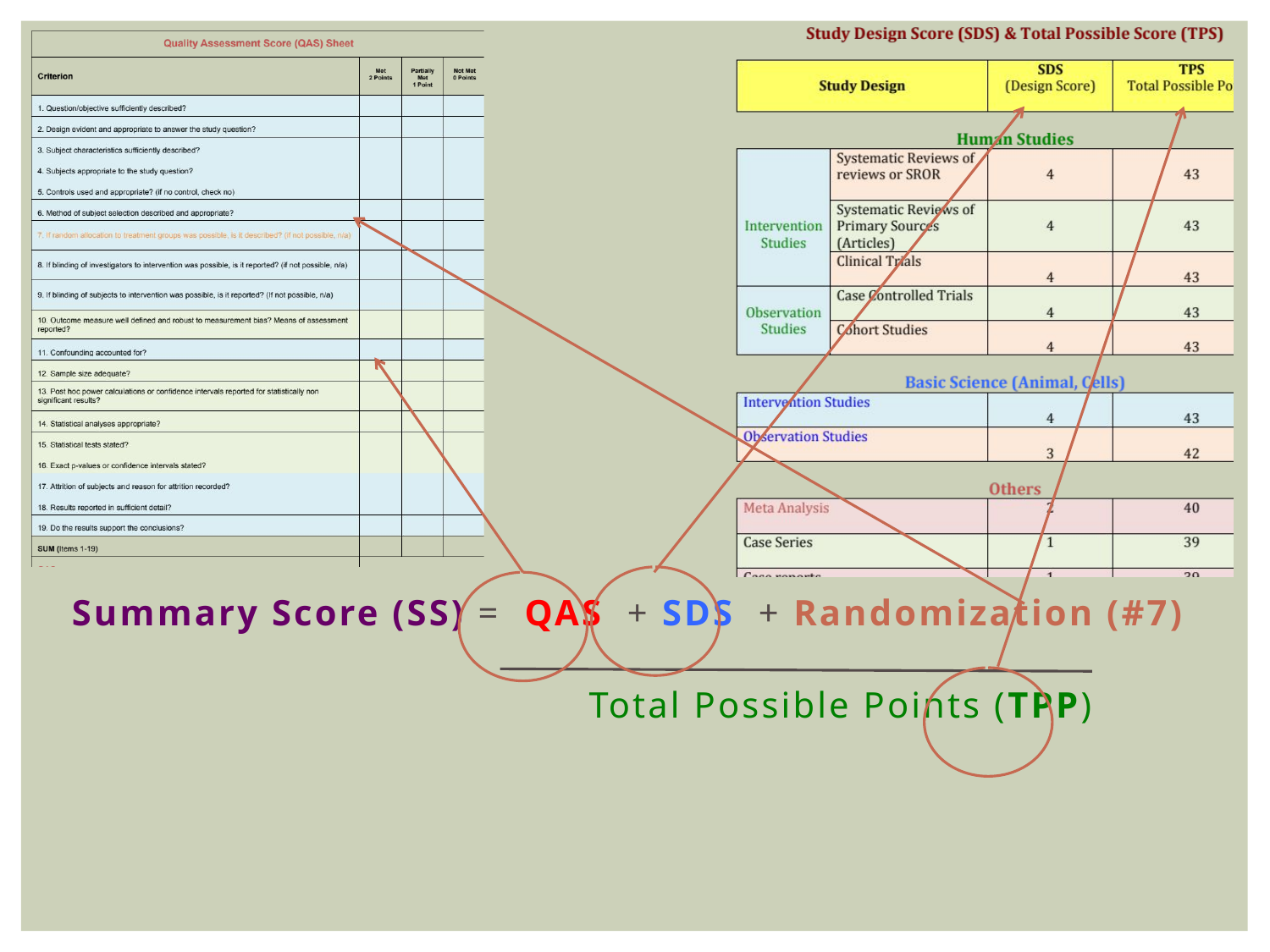

Summary Score (SS) = QAS + SDS + Randomization (#7)
 Total Possible Points (TPP)

## Slide 11
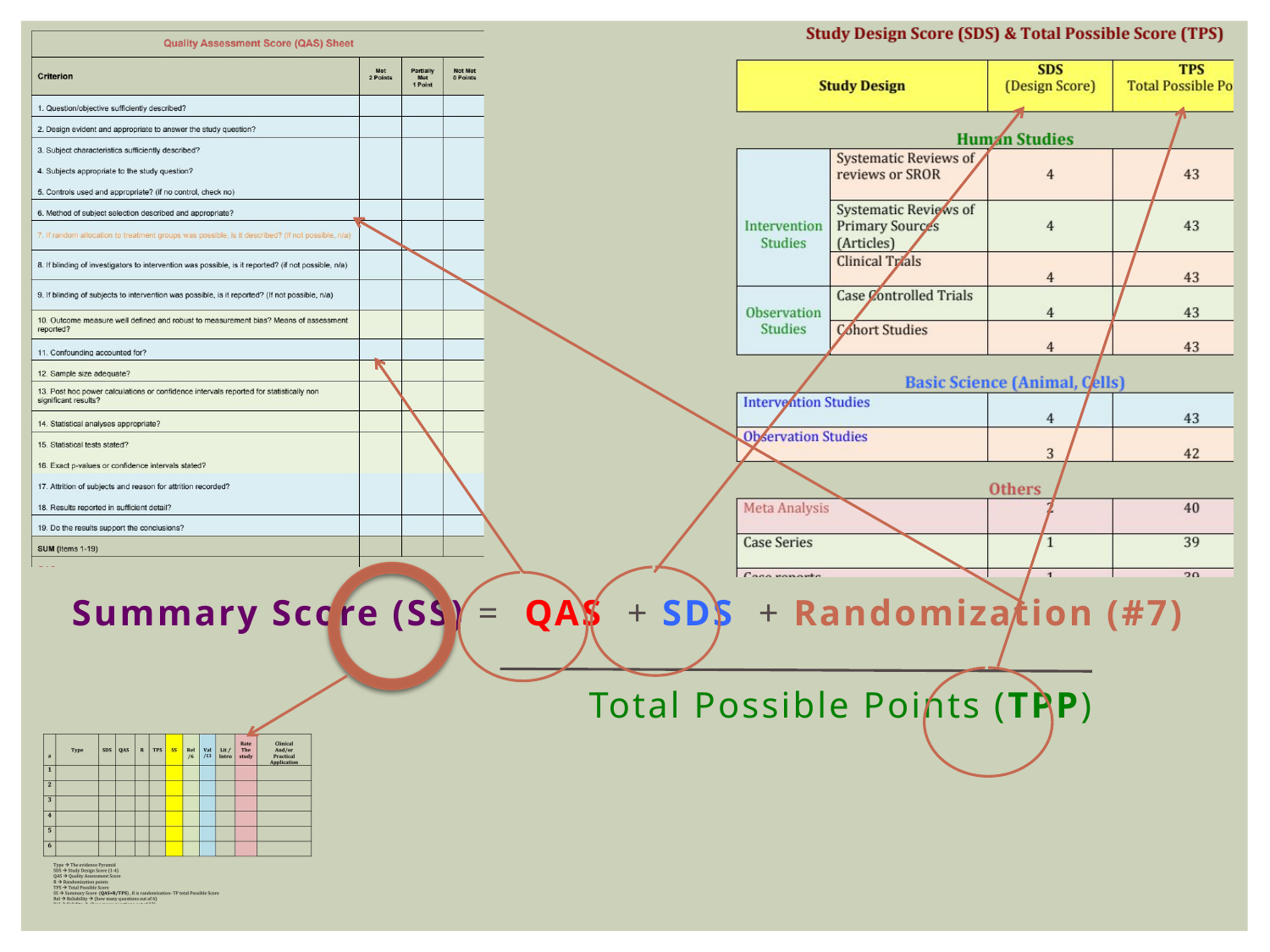

Summary Score (SS) = QAS + SDS + Randomization (#7)
 Total Possible Points (TPP)

## Slide 12
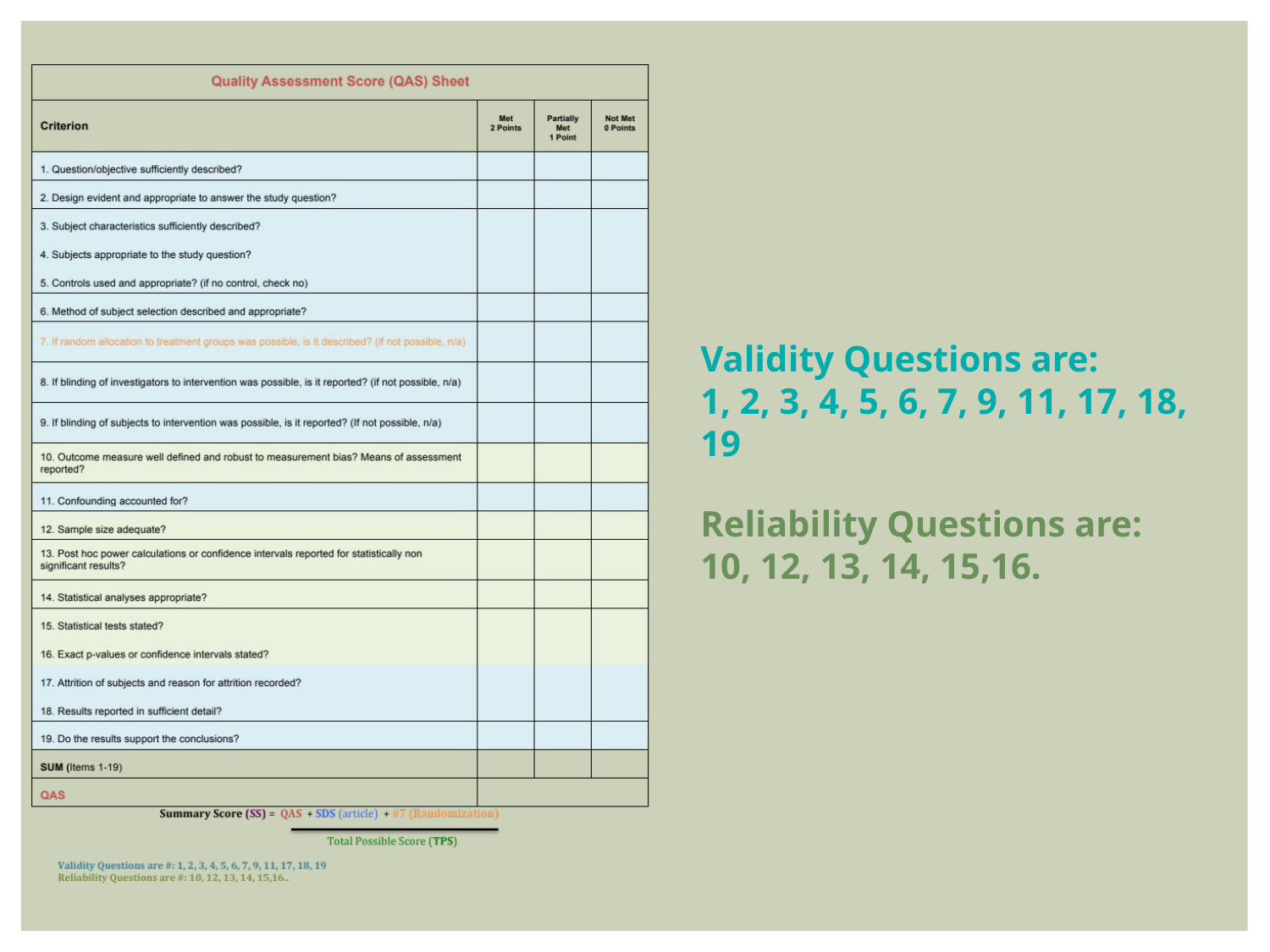

Validity Questions are:
1, 2, 3, 4, 5, 6, 7, 9, 11, 17, 18, 19
Reliability Questions are:
10, 12, 13, 14, 15,16.

## Slide 13
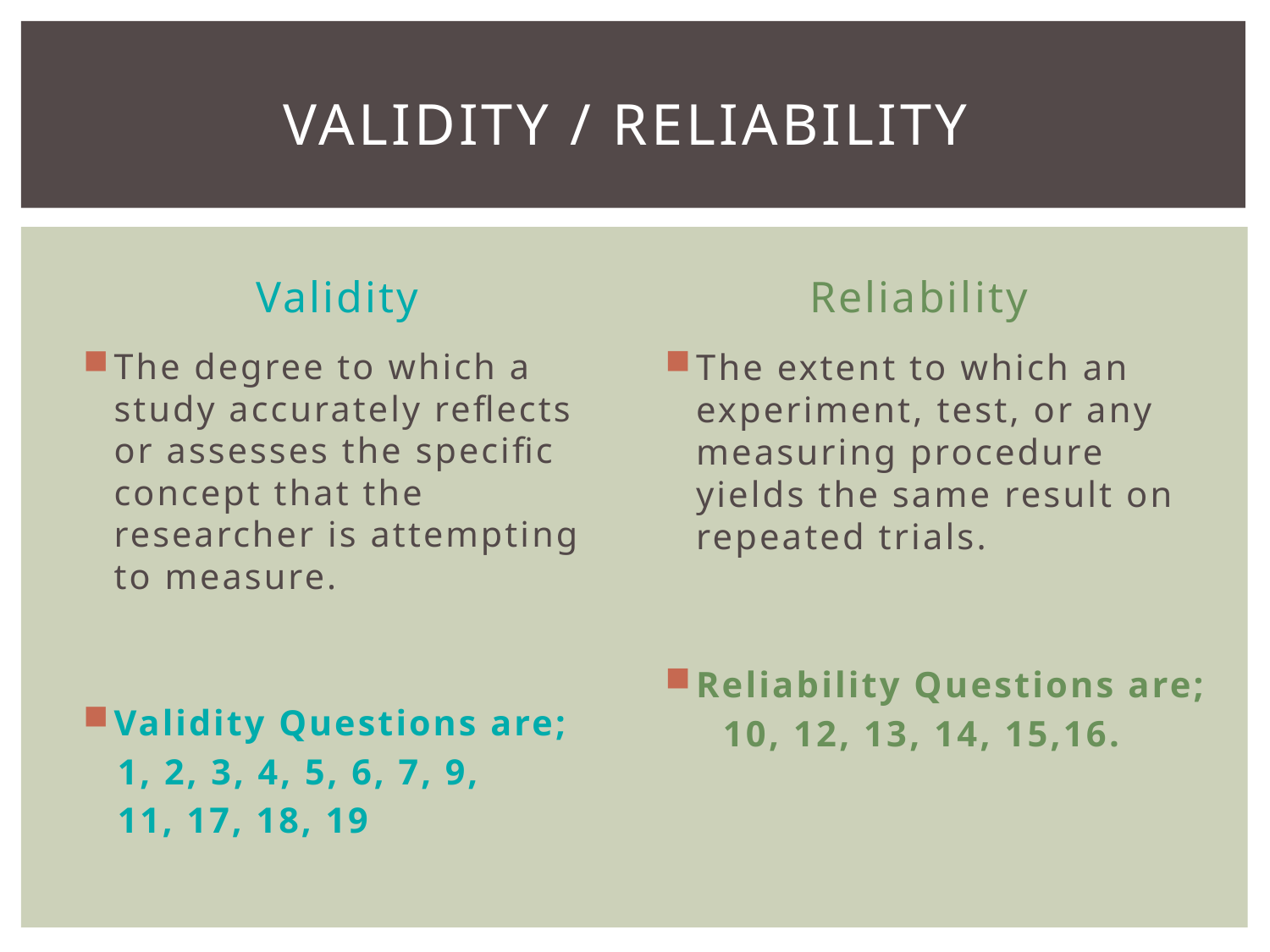

# Validity / Reliability
Validity
Reliability
The degree to which a study accurately reflects or assesses the specific concept that the researcher is attempting to measure.
Validity Questions are;
 1, 2, 3, 4, 5, 6, 7, 9,
 11, 17, 18, 19
The extent to which an experiment, test, or any measuring procedure yields the same result on repeated trials.
Reliability Questions are;
 10, 12, 13, 14, 15,16.

## Slide 14
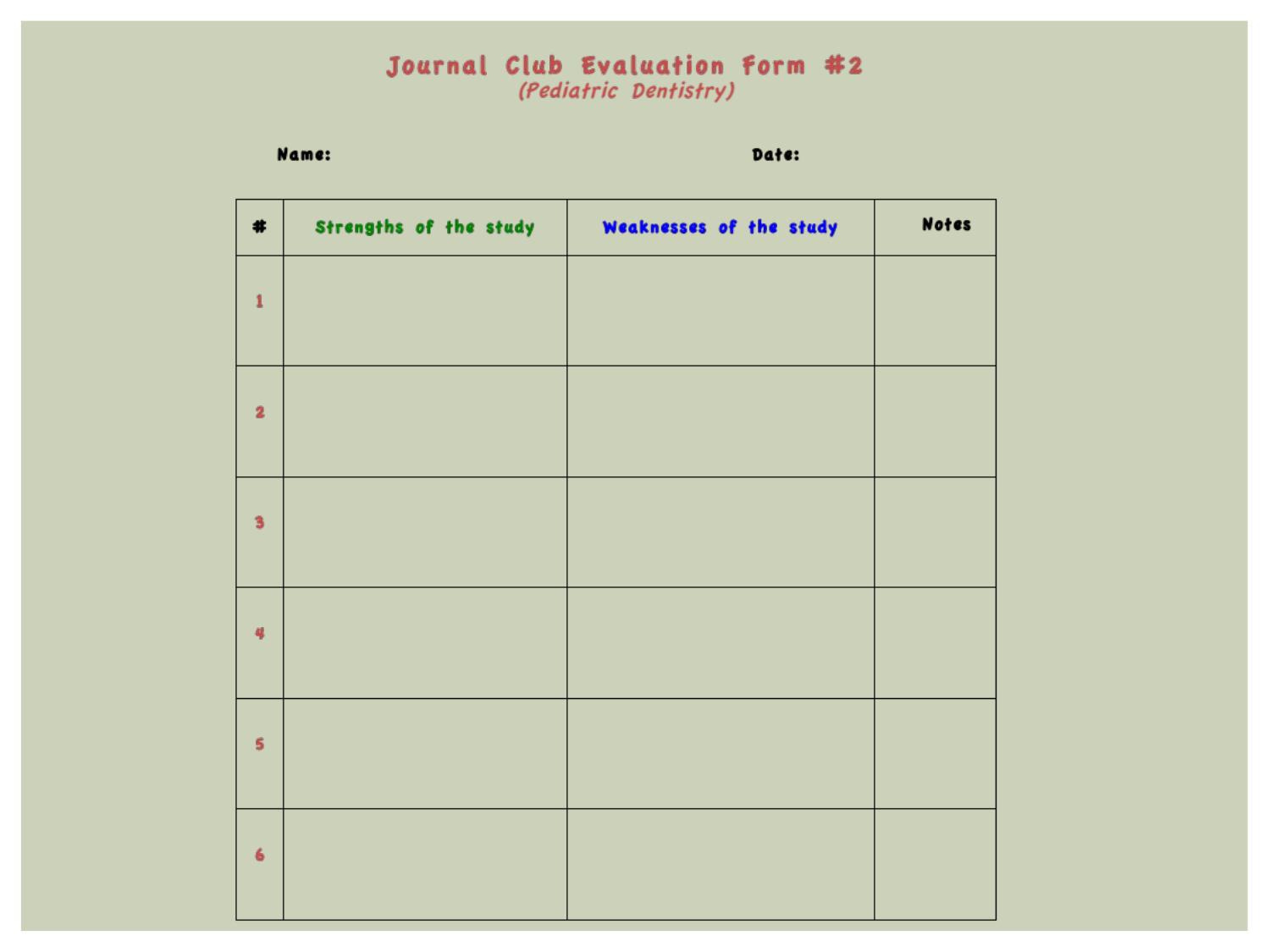

## Slide 15
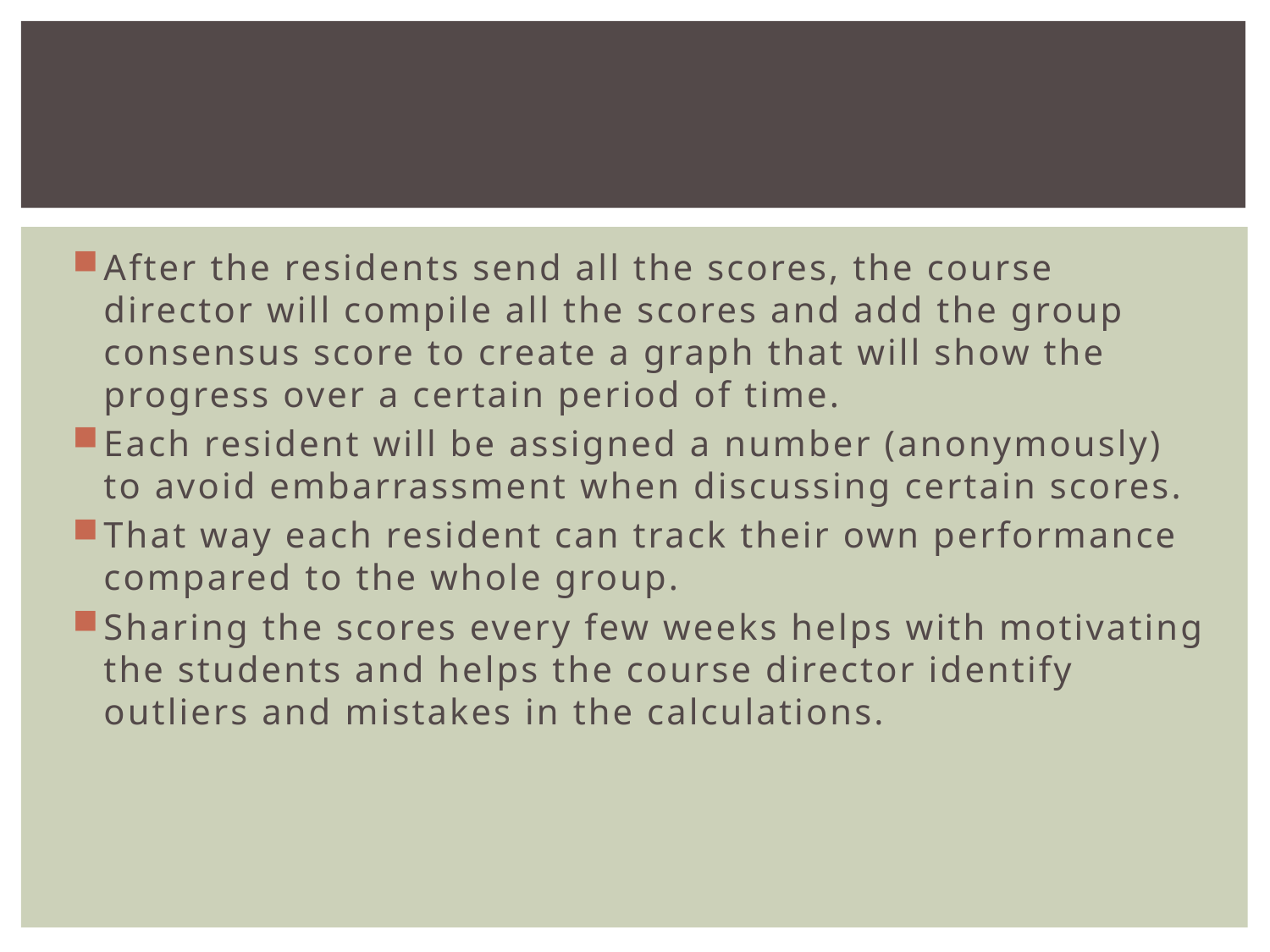

#
After the residents send all the scores, the course director will compile all the scores and add the group consensus score to create a graph that will show the progress over a certain period of time.
Each resident will be assigned a number (anonymously) to avoid embarrassment when discussing certain scores.
That way each resident can track their own performance compared to the whole group.
Sharing the scores every few weeks helps with motivating the students and helps the course director identify outliers and mistakes in the calculations.

## Slide 16
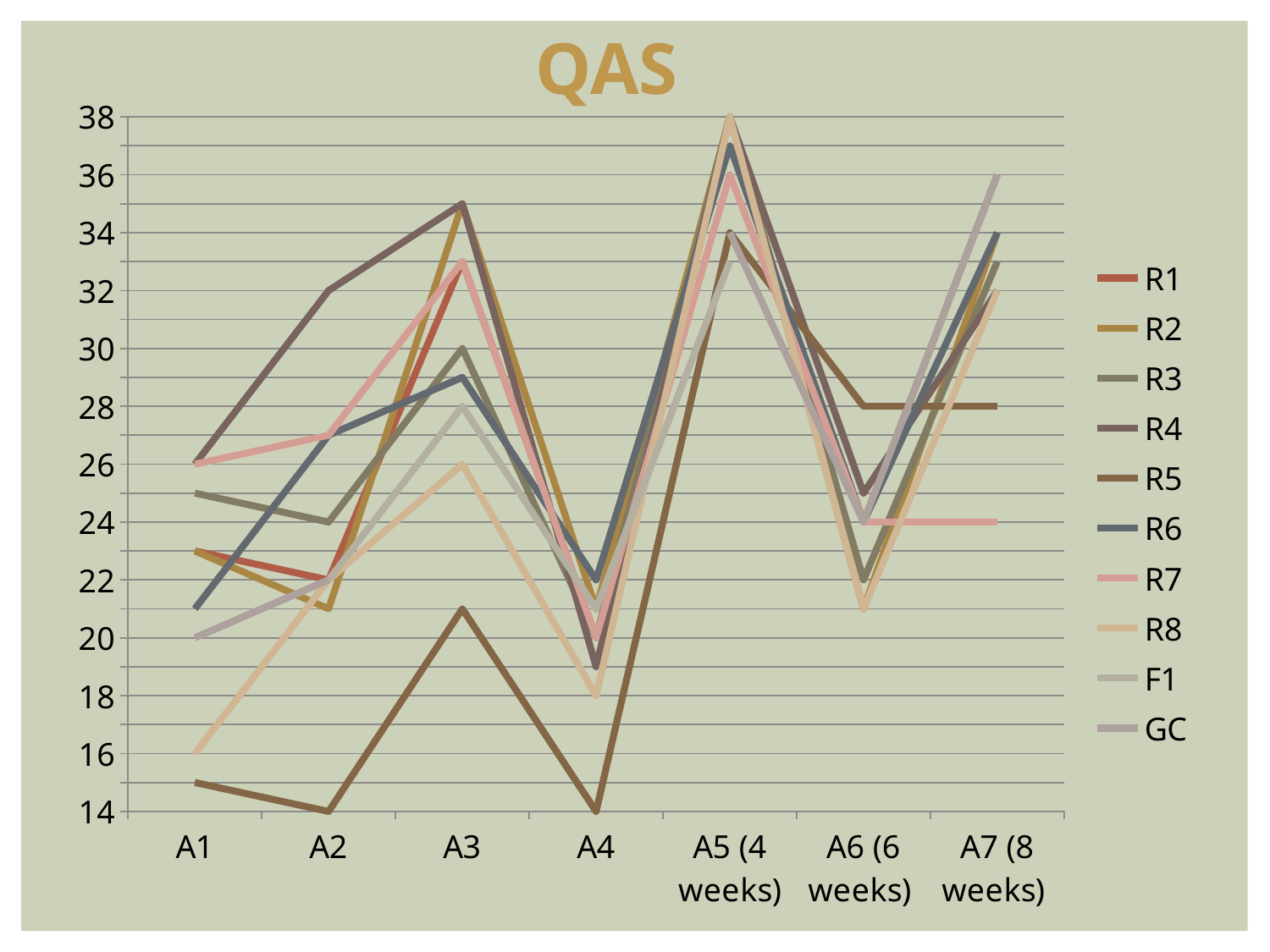

QAS
### Chart
| Category | R1 | R2 | R3 | R4 | R5 | R6 | R7 | R8 | F1 | GC |
|---|---|---|---|---|---|---|---|---|---|---|
| A1 | 23.0 | 23.0 | 25.0 | 26.0 | 15.0 | 21.0 | 26.0 | 16.0 | 20.0 | 20.0 |
| A2 | 22.0 | 21.0 | 24.0 | 32.0 | 14.0 | 27.0 | 27.0 | 22.0 | 22.0 | 22.0 |
| A3 | 33.0 | 35.0 | 30.0 | 35.0 | 21.0 | 29.0 | 33.0 | 26.0 | 28.0 | None |
| A4 | 20.0 | 21.0 | 20.0 | 19.0 | 14.0 | 22.0 | 20.0 | 18.0 | 21.0 | None |
| A5 (4 weeks) | None | 38.0 | 38.0 | 38.0 | 34.0 | 37.0 | 36.0 | 38.0 | 33.0 | 34.0 |
| A6 (6 weeks) | 24.0 | 21.0 | 22.0 | 25.0 | 28.0 | 24.0 | 24.0 | 21.0 | None | 24.0 |
| A7 (8 weeks) | 36.0 | 34.0 | 33.0 | 32.0 | 28.0 | 34.0 | 24.0 | 32.0 | None | 36.0 |

## Slide 17
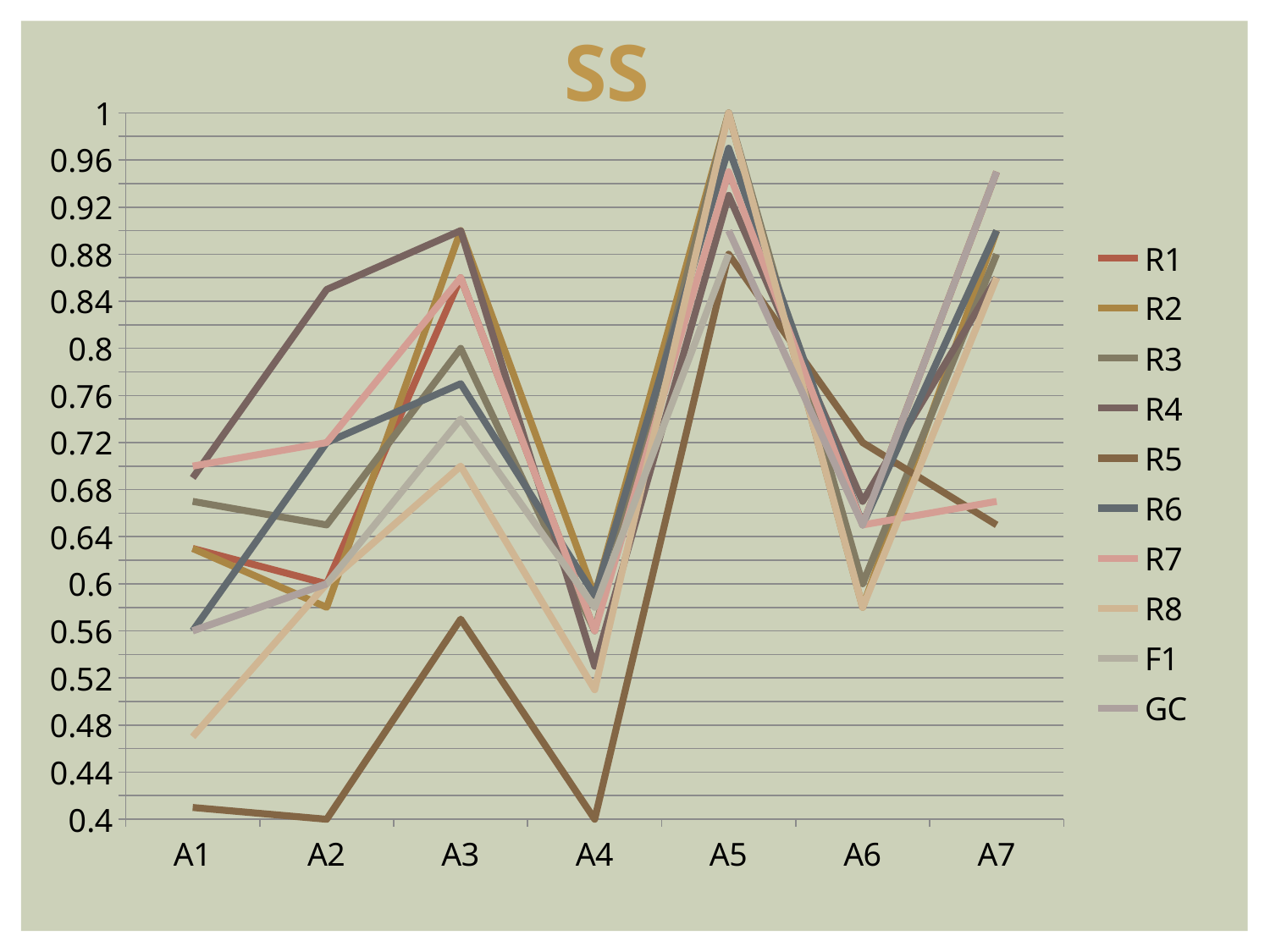

SS
### Chart
| Category | R1 | R2 | R3 | R4 | R5 | R6 | R7 | R8 | F1 | GC |
|---|---|---|---|---|---|---|---|---|---|---|
| A1 | 0.63 | 0.63 | 0.67 | 0.69 | 0.41 | 0.56 | 0.7 | 0.47 | 0.56 | 0.56 |
| A2 | 0.6 | 0.58 | 0.65 | 0.85 | 0.4 | 0.72 | 0.72 | 0.6 | 0.6 | 0.6 |
| A3 | 0.86 | 0.9 | 0.8 | 0.9 | 0.57 | 0.77 | 0.86 | 0.7 | 0.74 | None |
| A4 | 0.56 | 0.59 | 0.56 | 0.53 | 0.4 | 0.59 | 0.56 | 0.51 | 0.58 | None |
| A5 | None | 1.0 | 1.0 | 0.93 | 0.88 | 0.97 | 0.95 | 1.0 | 0.88 | 0.9 |
| A6 | 0.65 | 0.58 | 0.6 | 0.67 | 0.72 | 0.65 | 0.65 | 0.58 | None | 0.65 |
| A7 | 0.95 | 0.9 | 0.88 | 0.86 | 0.65 | 0.9 | 0.67 | 0.86 | None | 0.95 |

## Slide 18
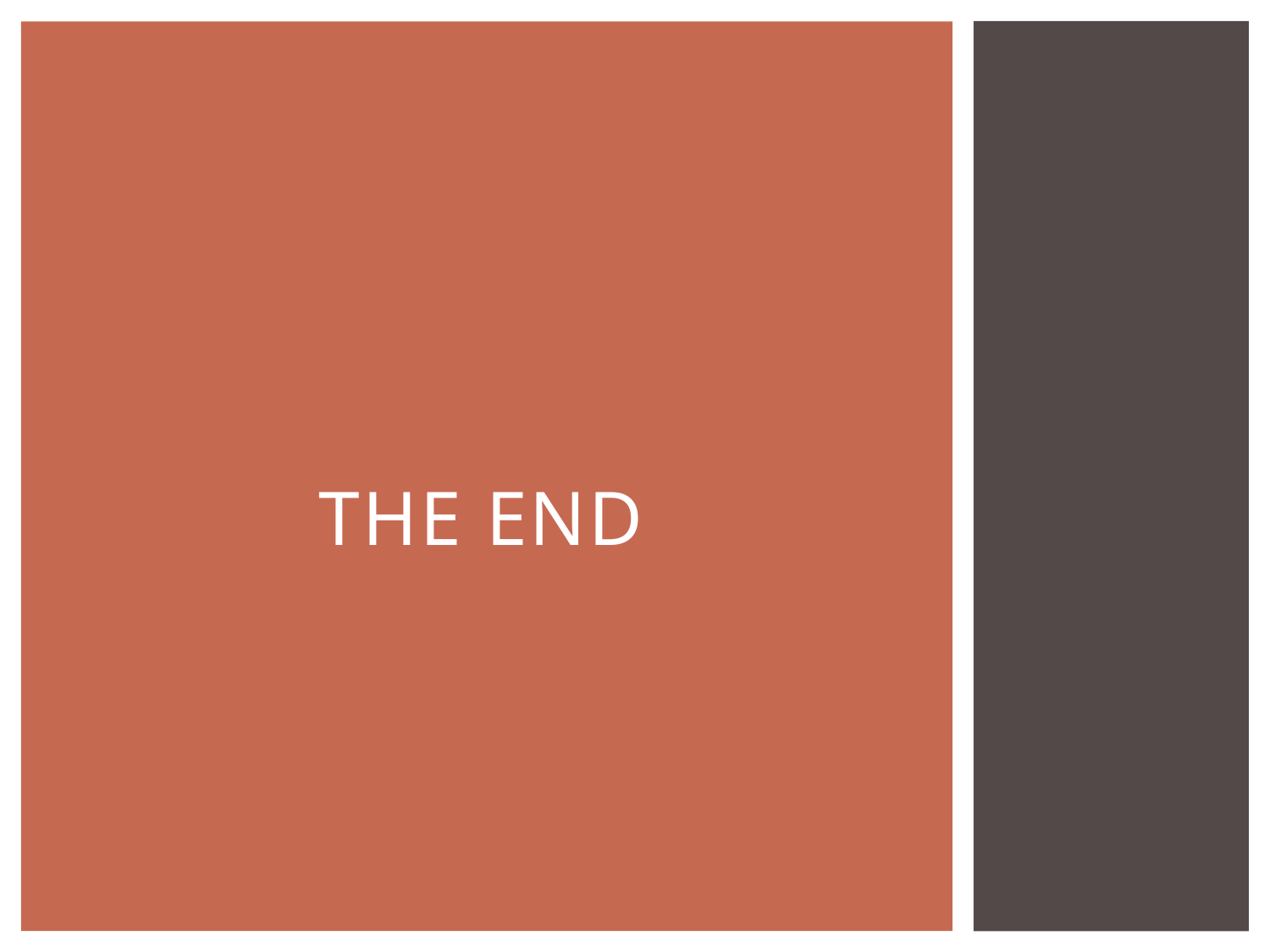

# The End
