# Supplementary material for: Using the Timmer Scale to Standardize Pediatric Dentistry Residents’ Scientific Appraisal Skills
Source: MedEdPORTAL. 2021 Feb 12;17:11101. doi: 10.15766/mep_2374-8265.11101 (PMC7880256; doi:10.15766/mep_2374-8265.11101)
Supplement: Supplementary file 1 — Introductory Course Material (EBP).pptxJournal Club Course Introduction.pptxQuality Assessment Score Sheet.docxStudy Design and Total Possible Points Form.docxArticles Evaluation Form.docxCourse Evaluation Form.docxPreclass and Remediation Reading Assignments.docx [file mep_2374-8265.11101-s001.zip › C. Quality Assessment Score Sheet.docx]

| **Quality Assessment Score (QAS) Sheet** | | | |
| --- | --- | --- | --- |
| **Criterion** | **Met**  **2 Points** | **Partially**  **Met**  **1 Point** | **Not Met**  **0 Points** |
| 1. Question/objective sufficiently described? |  |  |  |
| 2. Design evident and appropriate to answer the study question? |  |  |  |
| 3. Subject characteristics sufficiently described? |  |  |  |
| 4. Subjects appropriate to the study question? |  |  |  |
| 5. Controls used and appropriate? (if no control, check no) |  |  |  |
| 6. Method of subject selection described and appropriate? |  |  |  |
| 7. If random allocation to treatment groups was possible, is it described? (if not possible, n/a) |  |  |  |
| 8. If blinding of investigators to intervention was possible, is it reported? (if not possible, n/a) |  |  |  |
| 9. If blinding of subjects to intervention was possible, is it reported? (If not possible, n/a) |  |  |  |
| 10. Outcome measure well defined and robust to measurement bias? Means of assessment reported? |  |  |  |
| 11. Confounding accounted for? |  |  |  |
| 12. Sample size adequate? |  |  |  |
| 13. Post hoc power calculations or confidence intervals reported for statistically non significant results? |  |  |  |
| 14. Statistical analyses appropriate? |  |  |  |
| 15. Statistical tests stated? |  |  |  |
| 16. Exact p-values or confidence intervals stated? |  |  |  |
| 17. Attrition of subjects and reason for attrition recorded? |  |  |  |
| 18. Results reported in sufficient detail? |  |  |  |
| 19. Do the results support the conclusions? |  |  |  |
| **SUM (**Items 1-19) |  |  |  |
| **(QAS)** |  | | |
| **(SS) Summary Score (SS)** = **QAS** + **SDS** (article) + **#7 (Randomization)**  Total Possible Points (**TPP**) |  | | |

**Validity Questions are #: 1, 2, 3, 4, 5, 6, 7, 9, 11, 17, 18, 19 Reliability Questions are #: 10, 12, 13, 14, 15,16.**

Form is adapted from “Timmer A, Sutherland LR, Hilsden RJ. Development and evaluation of a quality score for abstracts. *BMC Med Res Methodol*. 2003;3(2).” Form is used with permission.
