# Supplementary material for: Using the Timmer Scale to Standardize Pediatric Dentistry Residents’ Scientific Appraisal Skills
Source: MedEdPORTAL. 2021 Feb 12;17:11101. doi: 10.15766/mep_2374-8265.11101 (PMC7880256; doi:10.15766/mep_2374-8265.11101)
Supplement: Supplementary file 1 — Introductory Course Material (EBP).pptxJournal Club Course Introduction.pptxQuality Assessment Score Sheet.docxStudy Design and Total Possible Points Form.docxArticles Evaluation Form.docxCourse Evaluation Form.docxPreclass and Remediation Reading Assignments.docx [file mep_2374-8265.11101-s001.zip › D. Study Design and Total Possible Points Form.docx]

**Study Design Score (SDS) & Total Possible Points (TPP)**

| **Study Design** | **SDS**  (Design Score) | **TPP**  (Total Possible Points) |
| --- | --- | --- |

**Human Studies**

| Intervention  Studies | Systematic Reviews of reviews or SROR | 4 | 43 |
| --- | --- | --- | --- |
|  | Systematic Reviews of Primary Sources (Articles) | 4 | 43 |
|  | Clinical Trials | 4 | 43 |
| Observation  Studies | Case Controlled Trials | 3 | 42 |
|  | Cohort Studies, prospective | 4 | 43 |
|  | Cohort Studies, retrospective | 3 | 42 |

**Basic Science (Animal, Cells)**

| Intervention Studies | 4 | 43 |
| --- | --- | --- |
| Observation Studies | 3 | 42 |

**Others**

| Meta Analysis | 2 | 40 |
| --- | --- | --- |
| Case Series | 1 | 39 |
| Case reports | 1 | 39 |
| Lab Studies (In Vitro) | 1 | 39 |
| Literature Review, Opinion, Expert Advise | 1 | 39 |
| Instrument Validation | 1 | 39 |
| Others / Do not know / Not sure | 0 | 38 |

*** TPP = Total Possible Points QAS + Randomization**

Form adapted from “Timmer A, Sutherland LR, Hilsden RJ. Development and evaluation of a quality score for abstracts. *BMC Med Res Methodol*. 2003;3(2).” Form is used with permission.
