# Supplementary material for: Using the Timmer Scale to Standardize Pediatric Dentistry Residents’ Scientific Appraisal Skills
Source: MedEdPORTAL. 2021 Feb 12;17:11101. doi: 10.15766/mep_2374-8265.11101 (PMC7880256; doi:10.15766/mep_2374-8265.11101)
Supplement: Supplementary file 1 — Introductory Course Material (EBP).pptxJournal Club Course Introduction.pptxQuality Assessment Score Sheet.docxStudy Design and Total Possible Points Form.docxArticles Evaluation Form.docxCourse Evaluation Form.docxPreclass and Remediation Reading Assignments.docx [file mep_2374-8265.11101-s001.zip › E. Articles Evaluation Form.docx]

**Journal Club Evaluation Form**

*(Pediatric Dentistry)*

**Residents Name: Date:**

| **Number of the Article** | **Strengths of the study** | **Weaknesses of the study** | **Notes** |
| --- | --- | --- | --- |
| **1** |  |  |  |
| **2** |  |  |  |
| **3** |  |  |  |
| **4** |  |  |  |
| **5** |  |  |  |
| **6** |  |  |  |
