# Supplementary material for: Using the Timmer Scale to Standardize Pediatric Dentistry Residents’ Scientific Appraisal Skills
Source: MedEdPORTAL. 2021 Feb 12;17:11101. doi: 10.15766/mep_2374-8265.11101 (PMC7880256; doi:10.15766/mep_2374-8265.11101)
Supplement: Supplementary file 1 — Introductory Course Material (EBP).pptxJournal Club Course Introduction.pptxQuality Assessment Score Sheet.docxStudy Design and Total Possible Points Form.docxArticles Evaluation Form.docxCourse Evaluation Form.docxPreclass and Remediation Reading Assignments.docx [file mep_2374-8265.11101-s001.zip › F. Course Evaluation Form.docx]

**Pediatrics Dentistry Department**

**Course Evaluation**

**Journal Club and Case Presentations**

**Course Director: ___________________**

**Date: ______________**

**Lecturer/Faculty Evaluation**

| Please Indicate Your Score in Each Section with an "X"  1 is poor, 4 is excellent | 1 | 2 | 3 | 4 | N/A |
| --- | --- | --- | --- | --- | --- |
| 1. Lecturer has a thorough knowledge and understanding of material. |  |  |  |  |  |
| 2. Lecturer presents material in a clear and easy to understand manner. |  |  |  |  |  |
| 3.The Lecturer encourages student’s discussion and participation. |  |  |  |  |  |
| 4.The Lecturer responds effectively to students' comments/questions. |  |  |  |  |  |
| 5. The overall evaluation of this Lecturer. |  |  |  |  |  |

**Course Evaluation**

| Please Indicate Your Score in Each Section with an "X" | 1 | 2 | 3 | 4 | N/A |
| --- | --- | --- | --- | --- | --- |
| 1. Course activities are appropriate to learning outcome. |  |  |  |  |  |
| 2. Course material is organized with a clear schedule. |  |  |  |  |  |
| 3. The material contains relevant and updated information. |  |  |  |  |  |
| 4. This course has strengthened my knowledge of the subject. |  |  |  |  |  |
| 5.The course makes an important contribution to the program. |  |  |  |  |  |
| 6. The overall evaluation of this Course. |  |  |  |  |  |

Comments

___________________________________________________________________________________________________________

___________________________________________________________________________________________________________

___________________________________________________________________________________________________________

___________________________________________________________________________________________________________

Do you have any suggestions to improve the course?

___________________________________________________________________________________________________________

___________________________________________________________________________________________________________

___________________________________________________________________________________________________________

___________________________________________________________________________________________________________
