# Supplementary material for: Using the Timmer Scale to Standardize Pediatric Dentistry Residents’ Scientific Appraisal Skills
Source: MedEdPORTAL. 2021 Feb 12;17:11101. doi: 10.15766/mep_2374-8265.11101 (PMC7880256; doi:10.15766/mep_2374-8265.11101)
Supplement: Supplementary file 1 — Introductory Course Material (EBP).pptxJournal Club Course Introduction.pptxQuality Assessment Score Sheet.docxStudy Design and Total Possible Points Form.docxArticles Evaluation Form.docxCourse Evaluation Form.docxPreclass and Remediation Reading Assignments.docx [file mep_2374-8265.11101-s001.zip › G. Preclass and Remediation Reading Assignments.docx]

**Preclass Reading Articles**

1. Young A. Enhancing Patient Care Through Evidence-Based Dentistry. *CDA*. 2018;46(9):553-554.
2. Kirk D. Searching for Research Articles on the Internet. *CDA*. 2018;46(9):555-559.
3. Young A. The Anatomy of a Clinical Study. *CDA*. 2018;46(9):561-566.
4. Shaneyfelt T. How to Critically Appraise the Dental Literature. *CDA*. 2018;46(9):569-576.
5. Salmon E*.* Statistics for Practicing Dentists. *CDA*. 2018;46(9):577-581.

**Remediation Articles**

1. Brignardello-Petersen R, Carrasco-Labra A, Glick M, Guyatt GH, Azarpazhooh A. A practical approach to evidence-based dentistry: Understanding and applying the principles of EBD. *JADA*. 2014;145(11):1105-1107.
2. Brignardello-Petersen R, Carrasco-Labra A, Glick M, Azarpazhooh A. A practical approach to evidence-based dentistry: How to appraise and use an article about therapy. *JADA*. 2015;146(11):42-49.
3. Carrasco-Labra A, Brignardello-Petersen R, Glick M, Guyatt GH, Azarpazhooh A. A practical approach to evidence-based dentistry VI: How to use a Systematic Review. *JADA*. 2015;146(11):42-49.
